# Golgi-protein 73 facilitates vimentin polymerization in hepatocellular carcinoma

**DOI:** 10.7150/ijbs.85431

**Published:** 2023-07-16

**Authors:** Xinyang Hu, Shijin Yuan, Sining Zhou, Ting Sun, Chaoqun Wang, Shilong Ying, Heping Zhu, Jingfeng Luo, Hongchuan Jin, Yiming Liu

**Affiliations:** 1 Laboratory of Cancer Biology, Key Laboratory of Biotherapy of Zhejiang Province, Sir Run Run Shaw Hospital, Zhejiang University School of Medicine, Hangzhou 310016, China.; 2 Cancer Center, Zhejiang University, Hangzhou 310058, China.; 3 State Key Laboratory for Diagnosis and Treatment of Infectious Diseases, Collaborative Innovation Center for Diagnosis and Treatment of Infectious Disease, The First Affiliated Hospital, Zhejiang University School of Medicine, Hangzhou 310003, China.; 4 Department of Pathology, The First Affiliated Hospital, Zhejiang University School of Medicine, Hangzhou 310003, China.; 5 Department of Pathology, Affiliated Dongyang Hospital of Wenzhou Medical University, Dongyang 322100, China.; 6 Department of Chemistry, Zhejiang University, Hangzhou 310058, China.

**Keywords:** hepatocellular carcinoma, GP73, vimentin, Clomipramine, cancer metastasis

## Abstract

Golgi-protein 73 (GP73) is highly expressed in hepatocellular carcinoma (HCC) and, as a secretory protein, it has been proposed as a serum biomarker indicating progression of HCC. The underlying mechanism by which GP73 may promote HCC metastasis is still poorly understood. In this study, we discovered that GP73 interacted with vimentin to facilitate Serine/Threonine-protein phosphatase PP1-alpha (PP1A)-mediated dephosphorylation of vimentin at S56 and facilitated vimentin polymerization, which blocked vimentin degradation via TRIM56-mediated ubiquitin/proteasome-dependent pathway. Strikingly, Clomipramine, a 5-hydroxytryptamine receptor (5-HTR) agonist approved for the treatment of depression, impaired GP73-mediated vimentin polymerization to effectively inhibit metastasis of HCC with high GP73 expression, which provided a new strategy against HCC metastasis. Lastly, it was found that serum GP73 (sGP73) correlated positively with vimentin in primary tissues of HCC, suggesting that sGP73 might serve as a potential serum biomarker for companion diagnosis of HCC with highly expressed vimentin. In summary, this study reveals the process of GP73-mediated vimentin polymerization and proves that Clomipramine serves as a potential drug targeting vimentin for metastatic HCC patients with high sGP73 level.

## Introduction

Hepatocellular carcinoma (HCC) is the sixth common carcinoma with the fourth leading cause of cancer-related death worldwide^1^. Hepatitis B virus (HBV) or hepatitis C virus (HCV) infection is the main causes of HCC, which accounts for 80% of HCC cases, especially in China and other Asian countries^1^-^3^. HCC is one of the most lethal cancers with limited treatment options owing to the high metastatic potential.

Golgi-protein 73 (GP73, encoded by *GOLM1*) is a *cis*-Golgi-resident membrane protein, which is highly expressed in pathological tissues of HCC and detectable in serum derived from HCC patients^4^. Due to its high sensitivity and specificity in cancer diagnostics, serum GP73 (sGP73) has been regarded as a sensitive biomarker for HCC diagnosis^5^, ^6^. Biochemical and clinical analyses demonstrate that upregulation of GP73 could promote epithelial-mesenchymal transition (EMT) of HCC cells and facilitate HCC metastasis^7^-^10^. In addition, GP73 plays an important role in mediating the trafficking of epidermal growth factor receptor (EGFR), programmed cell death-ligand1 (PD-L1), and matrix metalloproteinases (MMPs) such as MMP-2 and 7^8^, ^10^-^12^. Thus, it is deemed that GP73 might play critical roles in facilitating cancer metastasis through regulating EMT-associated factors.^13^. However, the detailed mechanisms remain poorly understood.

In the present study, we aimed to identify interacting proteins of GP73 in HCC and further investigate how GP73 facilitates metastasis of HCC. Besides, we attempted to search for new diagnostic options for metastatic HCC and explore novel drugs targeting HCC with GP73 upregulation.

## Methods

### Reagents

All chemicals, including MG132 (HY-13259), Ac-DEVD-CHO (HY-P1001), Brefeldin A (BFA, HY-16592), Cycloheximide (CHX, HY-12320), Withaferin A (WFA, HY-N2065), Clomipramine (HY-B0457A), Sertraline (HY-B0176A), Thioridazine (HY-B0965A), Fluorescein 5-isothiocyanate (FITC, HY-66019), and Sorafenib (HY-10201) were purchased from MedChemExpress (Monmouth Junction, NJ, USA). Sepharose 4B beads (4B200) were purchased from Sigma-Aldrich Co. (St. Louis, MO, USA).

### Cell culture

MHCC-97H and HCC-LM3 cells were from the Liver Cancer Institute (Zhongshan Hospital, Fudan University, China); SK-Hep-1, HepG2, Hep3B, PLC and 293 T cells were from the American Type Culture Collection (ATCC, Manassas, VA, USA); L02, HepG2.2.15 and Huh-7 cells were from National Collection of Authenticated Cell Cultures (NCACC, Shanghai, China). Cells were cultured in RPMI-1640 (L02 cells) or DMEM (other cell lines) medium (Thermo Fisher, Carlsbad, CA, USA) supplemented with 10% fetal bovine serum (FBS, Thermo Fisher) in 5% CO2 at 37 °C. Cell lines above were authenticated by STR profiling at Cobioer Bioscience Co., Ltd. (Nanjing, China) and experiments were performed within < 10 passages after authentication.

### Identification of GP73-interacting proteins using mass spectrum

MHCC-97H cells cultured in 225 cm^2^ flask were transfected with pCMV3-c-FLAG or pCMV3-GP73-c-FLAG fusion vector, and fusion protein interacted proteins were purified using Anti-FLAG M2 Affinity Gel or Rabbit-IgG Agarose (Sigma-Alderich Co.) following manufacturer's instructions. Purified proteins were separated using SDS-PAGE and gels were silver stained using a fast silver staining kit (Beyotime, Nanjing, China) following manufacturer's instructions. Protein bands were cropped and digested. Peptide fragments were lyophilized and resuspended in 0.05% TFA solution. Then they were identified using Triple-time-of-flying 5600 mass spectrum (Triple-TOF, SCIEX, Redwood City, CA, USA). Data were searched based on UniProt database (Human). Proteins were filtered as unused ≥1.3, peptide probability >95.0%, protein probability >99.0%.

### Collection and treatment of clinical specimens

Primary tumor tissues and adjacent liver tissues derived from HCC patients in cohort 1 were from the Department of Pathology and Clinical Laboratory of Sir Run Run Shaw Hospital, Zhejiang University School of Medicine (SRRSH, Hangzhou, China) during 2020-2021. Serum samples of corresponding patients above were derived from Clinical Laboratory of SRRSH. Samples in cohort 2 were from SRRSH during 2022.

In this study, HCC patients were sorted as the 8^th^ edition staging system of the American Joint Committee on Cancer (AJCC) for HCC. The levels of GP73 and vimentin in pathological HCC and adjacent liver tissues were examined using immunohistochemical analysis as previously reported^10^. Besides, the expression of tissue vimentin was divided into high and low groups according to the IHC score 7 as the cutoff value, with IHC score < 7 considered as low expression group, and IHC score ≥ 7 considered as high expression group. The level of sGP73 was determined using a human GOLM1/GP73 ELISA kit (Raybiotech, Norcross, GA, USA), following the manufacturer's instructions. Details of HCC patient information are shown in Table S1.

### Gel filtration of vimentin polymers

Cells were lysed using 1×IP lysis buffer (Thermo Fisher) with a protease and phosphatase inhibitor cocktail (Thermo Fisher) for 30 m and centrifuged at 15,000×*g* for 15 m to remove debris. The gel filtration column (Cytiva Superdex™ 200, Thermo Fisher) was pretreated with cold PBS. Cell lysates were passed over the column at the speed of 0.5 mL/m. Fractions were collected every 10 drops per tube and analysed using co-IP followed by immunoblotting. Molecular mass of each component was determined using a Gel Filtration Calibration Kit HMW (GE Healthcare, Pittsburgh, PA, USA).

### Live-cell imaging of GP73/vimentin

For observation of real-time movements of GP73 and vimentin, 293T cells cultured in 28.2 mm glass bottom cell culture dishes (Nest, Wuxi, China) were co-transfected with pCMV3-GP73-GFP and pCMV3-vimentin-OFP for 48 h. Fusion proteins were tracked using a structural illumination microscope (SIM, Nikon Corporation, Japan) with excitations of 488 nm and 561 nm. Images were captured every 5 s for 5 m. Movement of GP73 particles were marked with yellow arrows.

### *In vitro* pull-down analysis of Clomipramine and vimentin

Clomipramine was linked to Sepharose 4B beads as shown in Fig. S5E. Whole cell lysate of MHCC-97H was prepared as described in *co-immunoprecipitation analysis*. Exogenous vimentin was prepared in 293T cells transfected with pCMV3-vimentin-c-FLAG vector and it was purified using an Anti-FLAG M2 Affinity Gel. Whole cell lysate (2 mg) or vimentin-FLAG protein (5 μg) was incubated with Clomipramine-Sepharose 4B or Sepharose 4B alone in the reaction buffer (50 mM Tris-HCl pH=7.5, 5 mM EDTA, 150 mM NaCl, 1 mM DTT, 0.01% NP-40, 2 μg/mL BSA and protease and phosphatase inhibitor cocktail) overnight at 4℃. The beads were washed for 6×10 m with washing buffer (50 mM Tris-HCl pH=7.5, 5 mM EDTA, 150 mM NaCl, 1 mM DTT, 0.01% NP-40), and then Clomipramine binding proteins were collected using 1×protein loading buffer. Protein signal was detected using immunoblotting with anti-vimentin antibodies.

### Molecular docking

The tetramer structure of coil1B domain of vimentin was obtained from PDB database (PDB accession number: 5WHF). To explore the accurate binding model for the clomipramine binding pocket of vimentin, we performed molecular docking analysis using the AutoDock Vina software based on the crystal structure analyzed by X-ray diffraction. Structures with RMSD<2 were selected. The precise model was determined and validated using gel filtration and mapping of the binding sites of vimentin/TRIM56 *in vitro*.

### *In vivo* metastasis and tumor growth assays

For *in vivo* metastasis assay, BALB/c-nude mice (4 weeks old, female, from Slac Laboratories, Shanghai) were housed in specific pathogen-free cages. The single blind method was adopted in the experiments. Nude mice were randomized (n=6, calculated using MedCalc Software; 3 mice per cage; solvent group was considered as control group) 3 d after acclimatization and *in vivo* metastasis models were generated by injecting Huh-7 (2×10^6^) or MHCC-97H (1×10^6^) cells via the tail vein. Animals were intraperitoneally injected with Clomipramine every other day immediately after cell injection. They were anesthetized using pentobarbital sodium and sacrificed 90 (Huh-7) or 70 (MHCC-97H) d after injected with Clomipramine and their lungs were excised for imaging, weighting and haematoxylin and eosin (H&E) staining. Toxicity of Clomipramine was assessed via measuring weight of mice. For *in vivo* tumor growth assay, BALB/c-nude mice (4 weeks old, female) were randomized (n=6, calculated using MedCalc Software; 3 mice per cage; solvent group was considered as control group) 3 d after acclimatization and xenograft models were generated by subcutaneously injecting Huh-7 (5×10^6^) or MHCC-97H (4×10^6^) cells. Animals were intraperitoneally injected with Clomipramine every other day after carcinogenesis. They were anesthetized using pentobarbital sodium and sacrificed 28 (Huh-7) or 20 (MHCC-97H) d after cell injection (To promise tumor volumes ≤1000 mm^3^, tumor volumes were measured using the formula: volume (mm^3^)=length×width×width/2. Toxicity of Clomipramine or Sorafenib was assessed via measuring weight of mice.

### Ethics statement

All clinical specimens were collected with the informed consent of the patients, and the experiments were approved by the Research Ethics Committee of SRRSH. All procedures for animal care and use were in compliance with the Guide for the Care and Use of Laboratory Animals (NIH, 8^th^ edition) and approved by the Institutional Animal Care and Use Committee at Zhejiang University. The study was performed accordance with the Declaration of Helsinki.

### Statistical analysis

The enumeration data was presented as frequency and percentage, and its significance was analyzed by a Chi-square or Fisher's exact test. The normality of measurement data was tested by Shapiro-Wilk test. The measurement data with normal distribution was presented as mean ±standard deviation (SD), while the measurement data with abnormal distribution was presented as median and interquartile range (IQR). In comparisons of two groups, statistical analysis was performed by student's t-test for normally distributed data, and Mann-Whitney U test for abnormally distributed data, respectively. In comparisons of three or more groups, statistical analysis was performed by one-way analysis of variance (ANOVA) for normally distributed data, and Kruskal-Wallis test for abnormally distributed data, respectively. Correlation analysis and coefficients were computed by Spearman method for abnormally distributed data.

The best cutoff values of each research variable were calculated by the “survcutpoint” function in the “survminer” R package. The survival curves for the prognostic analysis were conducted via the Kaplan-Meier method, and log-rank tests were used to judge significant differences between groups. The area under the receiver operating characteristic curve (ROC) was evaluated by “pROC” R package.

All statistical *P*-values were two-sided, with *P*-value < 0.05 considered as statistically significant. All statistical analyses were conducted using R 3.6.1.

## Results

### Vimentin is identified as a new GP73-interacting protein and regulated by GP73

**Figure 1:**
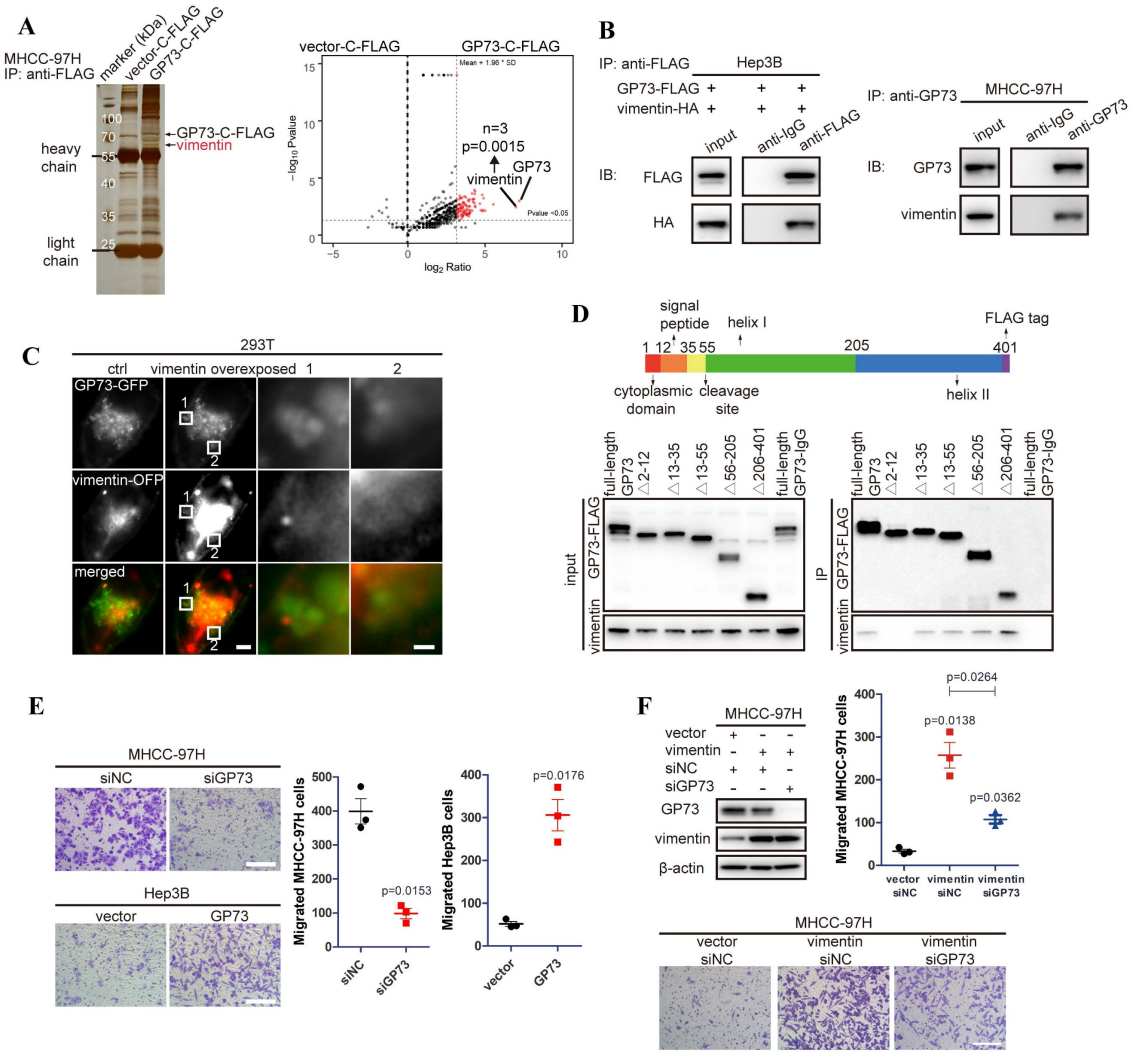
** Vimentin is identified as a new GP73-interacting protein facilitating HCC migration.** A. GP73-c-FLAG fusion protein overexpressed in MHCC-97H cells was purified with its interacted proteins for LC-MS/MS analysis and differential protein were shown by volcano map and vimentin was identified as a leading GP73-interacting protein via LC-MS/MS (n=3). B. Verification of the interaction of GP73 and vimentin in HCC cells via co-IP and immunoblotting analysis. C. Structural illumination microscopy of 293T cells expressing vimentin-OFP (red) and GP73-GFP (green, scale bar: 2 μm, left; 500 nm, right). D. The interactions of vimentin and GP73 truncated mutants were determined using co-IP followed by immunoblotting 72 h after 293T cells expressing GP73 truncated mutants. E. MHCC-97H and Hep3B cells were planked into Transwell chambers 48 h after the level of GP73 was modulated as indicated. Cells were cultured for extra 24 h, then, migrated cells were stained using crystal violet and counted (n=3, scale bar: 100 μm). F. The levels of GP73 and vimentin were determined in MHCC-97H cells transfected with siRNAs and vectors as described for 72 h. Transwell migration assay was conducted as above (n=3, scale bar: 100 μm). Data in A, E and F are the mean±s.e.m. and a two-tailed Student's *t*-test was used for statistical analysis.

To discover GP73-interacting proteins and investigate how GP73 facilitates metastasis of HCC, GP73-c-FLAG fusion protein was expressed in MHCC-97H cells, an HCC cell line with high metastatic ability. After co-immunoprecipitated (co-IP) using an anti-FLAG antibody followed by LC-MS/MS analysis, vimentin was identified as a leading GP73-interacting protein (Fig. 1A). Since Gene Ontology (GO) analysis further indicated that GP73-interacting proteins were enriched in cell migration and cell shape-associated signaling pathways (Fig. S1A), it was considered that GP73 might regulate vimentin, the leading GP73-interacting protein modulating cell migration and cell shape, to facilitate HCC migration. Independent co-IP and immunoblotting analyses further proved that GP73 interacted with vimentin in HCC cells (Fig. 1B).

To further confirm the interaction of GP73 with vimentin, GP73-GFP and vimentin-OFP fusion proteins were co-expressed in 293T cells. It showed that GP73 co-localized with vimentin on Golgi-originated vesicles (Fig. 1C), suggesting that GP73, as a Golgi-resident protein, might regulate the degradation, modification or polymerization of vimentin. Mapping of the binding sites of GP73 and vimentin demonstrated that vimentin interacted with GP73 in the region of cytoplasmic domain (Fig. 1D), implicating that vimentin might serve as a substrate of GP73-mediated trafficking^8^. Consistent with the critical roles of vimentin in promoting cell motility, GP73 knockdown inhibited migration of HCC cells, while opposite result was noticed with GP73 overexpression (Fig. 1E). Moreover, cell migration stimulated by vimentin overexpression was greatly impaired after GP73 knockdown (Fig. 1F). Knockdown of vimentin in GP73 overexpressed cells could also inhibit GP73-induced cell migration (Fig. S1B), confirming that the interaction of GP73 and vimentin is biologically relevant. Taken together, vimentin is identified as a new interacting partner of GP73.

**Figure 2:**
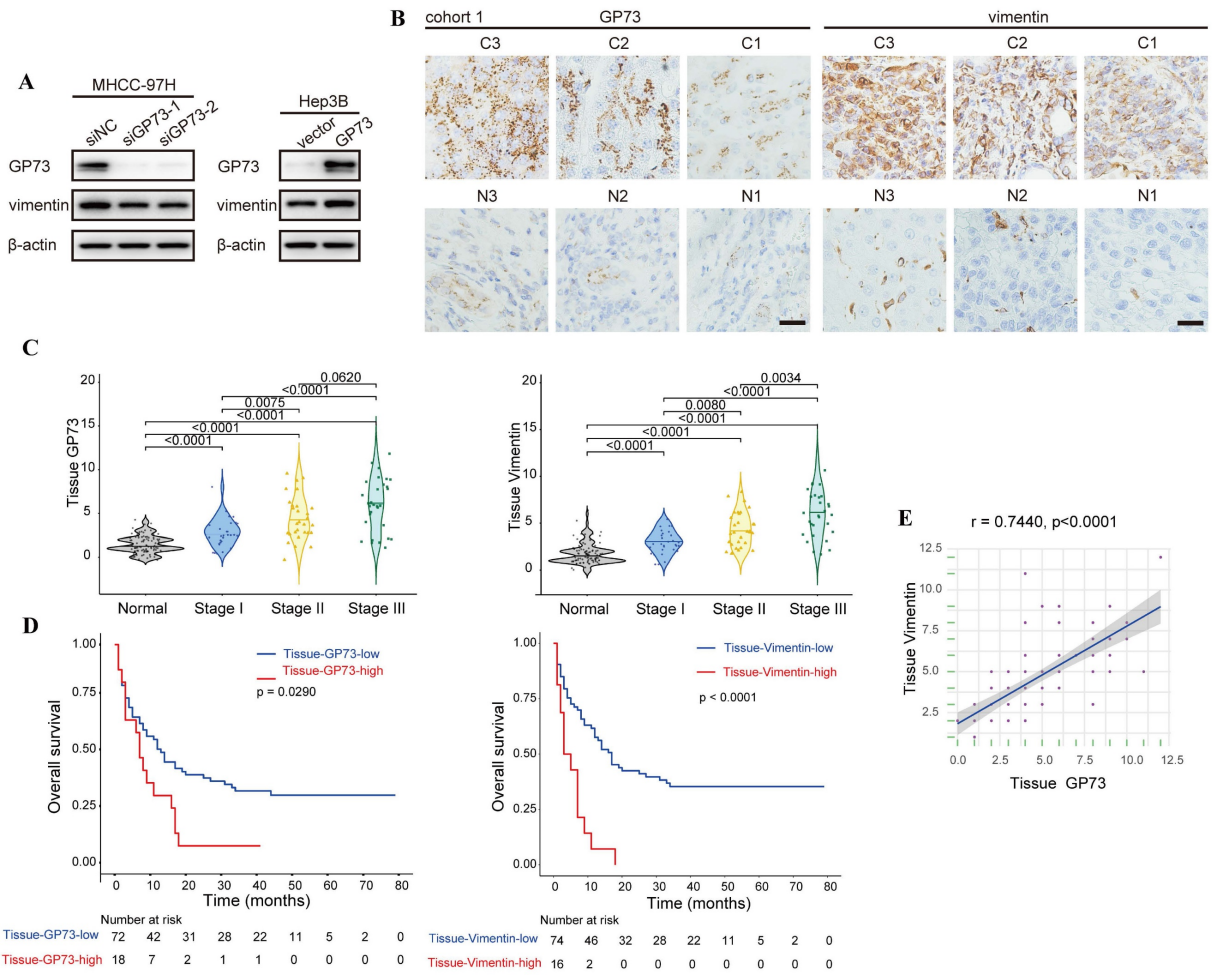
** GP73 positively regulates the expression of vimentin.** A. The levels of vimentin and GP73 were determined by immunoblotting analysis 72 h after the level of GP73 mediated in MHCC-97H and Hep3B cells. B. Immunohistochemical analysis of GP73 and vimentin in primary tumor (n=90) and adjacent liver (n=90) tissues of HCC patients. Images were labeled by staging of AJCC (1, 2 and 3) and tissue type (C=primary tumor, N=adjacent liver; scale bar: 15 μm). C. The levels of GP73 and vimentin were shown as IHC scoring. D. Overall survival of patients with high and low levels of GP73 and vimentin (n=90). E. The correlation of GP73 and vimentin in primary tumor was represented (n=90). Data in C are the mean±S.D. and one-way ANOVA was used for statistical analysis. Data in D are analyzed using log-rank tests and data in e are analyzed using spearman correlation analysis.

In an effort to demonstrate the co-localization of endogenous vimentin and GP73, we surprisingly noticed that the level of vimentin was reduced upon GP73 knockdown in MHCC-97H cells. In contrast, vimentin expression was increased after overexpressing GP73 in Hep3B cells (Fig. S2A). Immunoblotting analysis further confirmed that GP73 positively regulated the level of vimentin (Fig. 2A). Consistently, the levels of GP73 and vimentin in normal liver and HCC cell lines were positively correlated (r=0.7863, Fig. S2B). Next, immunohistochemical staining was performed to determine the levels of GP73 and vimentin in primary tumor tissues and corresponding adjacent liver tissues derived from HCC patients (n=90). It was manifested that both of them were highly expressed in primary tumor tissues in comparison with adjacent liver tissues (Fig. 2B and C). In addition, high expression of GP73 or vimentin was associated with short overall survival (OS) (Fig. 2D). Remarkably, the level of GP73 was significantly correlated with vimentin (r=0.7440, Fig. 2E). Thus, it was considered that GP73 increased the expression of vimentin.

### GP73 stabilizes vimentin protein by preventing TRIM56-mediated polyubiquitination

To investigate whether GP73 regulates the transcription of vimentin, the mRNA level of vimentin was measured in HCC cells with GP73 mediation. It showed that the mRNA level of vimentin was not significantly impacted upon GP73 knockdown or overexpression in HCC cells (Fig. S3A).

**Figure 3:**
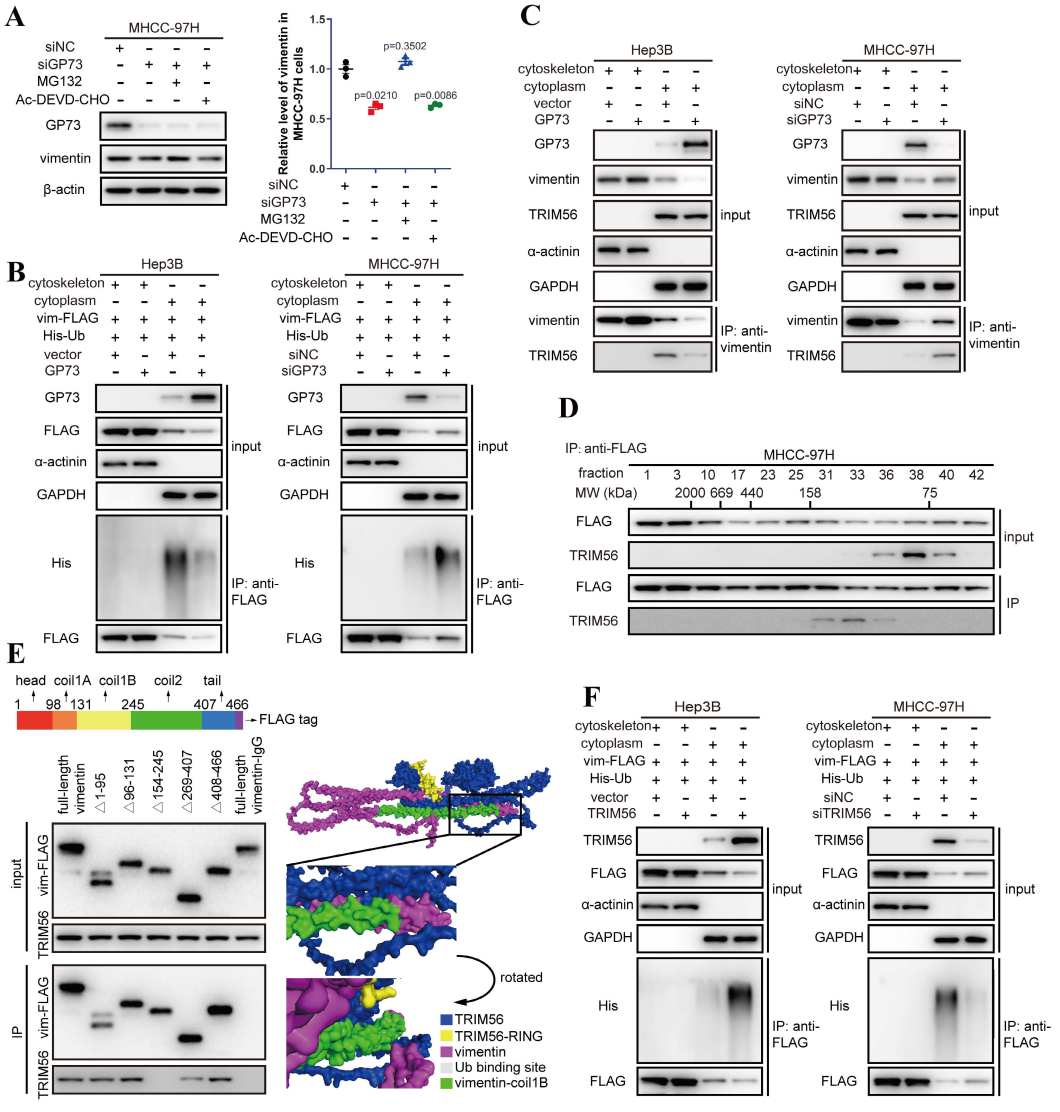
** GP73 stabilizes vimentin by preventing its polyubiquitination mediated by TRIM56.** A. The levels of GP73 and vimentin in MHCC-97H cells transfected with siNC or siGP73 were determined by immunoblotting. Cells were treated with MG132 (5 μM) or Ac-DEVD-CHO (100 μM) 12 h before harvest. B. Cytoskeletal and cytoplasmic components were isolated and the ubiquitination level of vimentin-FLAG in both components was determined by immunoblotting analysis 72 h after the level of GP73 mediated in MHCC-97H and Hep3B cells. C. Cytoskeletal and cytoplasmic components were isolated and the interaction of vimentin and TRIM56 was examined using co-IP followed by immunoblotting 72 h after the level of GP73 mediated in MHCC-97H and Hep3B cells. D. Components in whole cell lysates of MHCC-97H cells were separated by gel filtration as molecular weights (MWs) and the levels of vimentin and TRIM56 in each component were determined by immunoblotting analysis. E. The interactions of TRIM56 and vimentin truncated mutants were determined using co-IP followed by immunoblotting 72 h after 293T cells expressing vimentin truncated mutants. Predicted structure of vimentin-TRIM56 complex further verified the results. F. Cytoskeletal and cytoplasmic components were isolated and the ubiquitination level of vimentin-FLAG in both components was determined by immunoblotting analysis 72 h after the level of TRIM56 mediated in Hep3B and MHCC-97H cells. Data in A are the mean±s.e.m. and a two-tailed Student's *t*-test was used for statistical analysis.

Co-IP followed by immunoblotting demonstrated that the content of vimentin interacting with GP73 was consistent with purified GP73, which implied that GP73 was most likely to stabilize vimentin protein to increase its expression (Fig. S3B). Indeed, the half-life of vimentin protein was prolonged after GP73 overexpressing but shortened by GP73 knockdown (Fig. S3C), implicating that GP73 might prevent the degradation of vimentin. Since it has been reported that ubiquitin-proteasome pathway or caspase-3 mediates the degradation of vimentin^14^-^16^, the level of vimentin was examined in MHCC-97H^siGP73^ cells treated with inhibitors targeting proteasome or caspase-3. Knockdown of GP73 reduced the level of vimentin, which could be rescued by MG132 instead of Ac-DEVD-CHO (Fig. 3A), indicating that the reduction of vimentin after GP73 knockdown was most likely caused by ubiquitin-dependent proteasome degradation. Further study manifested that GP73 overexpression inhibited while GP73 knockdown enhanced poly-ubiquitination of vimentin (Fig. S3D), especially the cytoskeletal components (Fig. 3B).

To identify the E3 ubiquitin ligase involved in vimentin degradation, reported E3 ubiquitin ligases targeting vimentin were examined in HCC cells^14^, ^17^, ^18^. It revealed that knockdown of TRIM56 sharply attenuated the ubiquitination of vimentin (Fig. S3E), indicating that TRIM56 is potentially responsible for GP73-mediated vimentin degradation in HCC cells.

Interestingly, TRIM56 only interacted with vimentin in the cytoplasm where vimentin existed as monomers rather than polymers in the cytoskeletal fraction (Fig. 3C and Fig. S3F). It was echoed by co-IP followed by gel filtration, showing that TRIM56 interacted with vimentin monomers in the fractions where the molecular weight ranged from 75 to 158 kDa (Fig. 3D).

Mapping of the binding sites of vimentin and TRIM56 showed that TRIM56 interacted with vimentin in the region containing coilB and coil2 domain (Fig. 3E). Molecular docking further confirmed the necessity of coil1B domain to the interaction of vimentin and TRIM56 (Fig. 3E). It has been reported that vimentin polymerization is coil1B domain dependent^19^, indicating that the steric hinderance between coil1B domains in vimentin tetramers would block the interaction of TRIM56 and vimentin tetramers. Besides, it was supposed that TRIM56 might facilitate vimentin ubiquitination at K334, which was verified by co-IP analysis showing that TRIM56 failed to ubiquitinate vimentin-K334R mutant (Fig. S3G). Actually, TRIM56 could not interact with vimentin tetramers which were located in the cytoskeleton fraction (Fig. S3F). Much to our surprise, GP73 did not abrogate the interaction between vimentin and TRIM56, instead, GP73 overexpression reduced the amount of vimentin in the cytoplasm but increased its level in the cytoskeletal fraction, and vice versa (Fig. 3C). TRIM56 overexpression enhanced while TRIM56 knockdown inhibited poly-ubiquitination of cytoplasmic vimentin, further proving that TRIM56 was the very E3 ubiquitin ligase facilitating degradation of cytoplasmic vimentin (Fig. 3F).

Taken together, TRIM56 promotes ubiquitin-dependent proteasome degradation of vimentin in the cytoplasm, which was impaired by highly expressed GP73. Therefore, it is considered that GP73 might stabilize vimentin-mediated intermediate filaments through inhibiting vimentin degradation.

### GP73 promotes polymerization of vimentin to stabilize intermediate filament network

**Figure 4:**
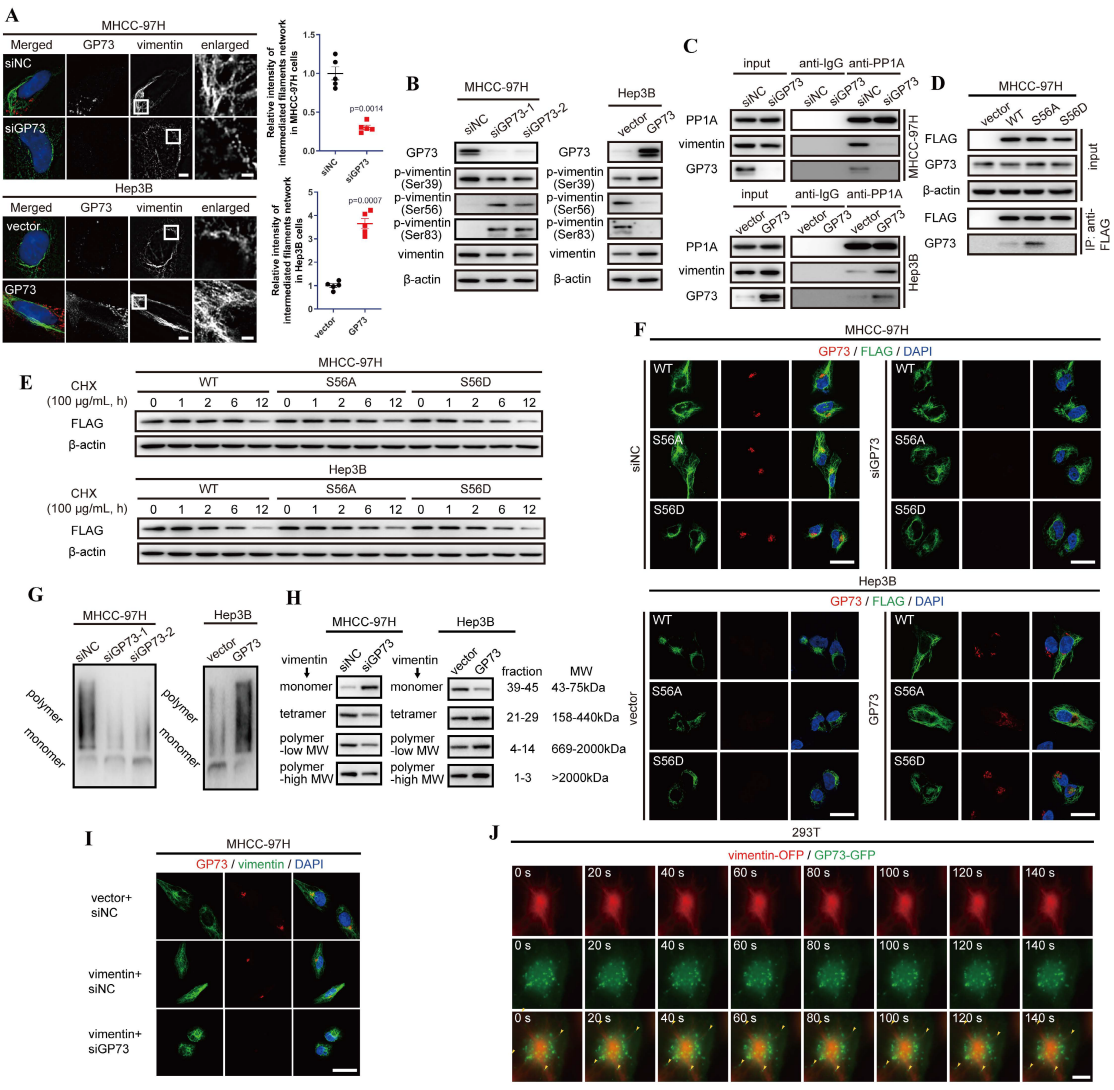
** GP73 promotes polymerization of vimentin to stabilize intermediate filament network.** A. Structural illumination microscopy of fine structure of vimentin (green) and GP73 (red) in MHCC-97H and Hep3B cells 48 h after the level of GP73 mediated (n=5, scale bar: 2 μm, left; 500 nm, right). B. The levels of p-vimentin (S39) and p-vimentin (S56) were determined by immunoblotting analysis 72 h after the level of GP73 mediated in MHCC-97H and Hep3B cells. C. The interactions of PP1A, vimentin and GP73 were determined using co-IP followed by immunoblotting 72 h after the level of GP73 mediated in MHCC-97H and Hep3B cells. D. The interactions of vimentin S56 mutants and GP73 were determined using co-IP followed by immunoblotting 72 h after MHCC-97H cells were transfected with indicated vectors. E. The levels of vimentin S56 mutants were determined by immunoblotting analysis after Hep3B and MHCC-97H cells were transfected with indicated vectors for 72 h. Cells were treated with CHX (100 μg/mL) for 0, 1, 2, 6 and 12 h before cells were harvested. F. Immunofluorescence staining of GP73 (red) and vimentin S56 mutants (FLAG-tag, green) 72 h after MHCC-97H and Hep3B cells were transfected with indicated vectors (scale bar: 10 μm). G. The polymerization degrees of vimentin in Hep3B and MHCC-97H cells were examined using Native-PAGE and immunoblotting assays. H. Components in whole cell lysates were separated by gel filtration as MWs and the levels of monomers, tetramers, low MW polymers and high MW polymers of vimentin were determined by immunoblotting analysis 72 h after the level of GP73 mediated in MHCC-97H and Hep3B cells. I. Immunofluorescence staining and confocal microscopy of vimentin (green) and GP73 (red) in MHCC-97H cells 72 h after the levels of GP73 and vimentin mediated (scale bar: 10 μm). J. Live-cell imaging of 293T cells expressing vimentin-OFP (red) and GP73-GFP (green). Images were captured by structural illumination microscope every 5 s for 5 m (scale bar: 5 μm). Data in A are the mean±s.e.m. and a two-tailed Student's *t*-test was used for statistical analysis.

To further investigate the effects of GP73 on vimentin polymerization, Structural illumination microscopy (SIM) was utilized to manifest the refined structures of vimentin-mediated intermediate filaments. It was revealed that overexpression of GP73 enhanced polymerization of vimentin to intermediate filament network while knockdown of GP73 depolymerized vimentin-mediated intermediate filaments (Fig. 4A). As it was shown that knockdown of GP73 gradually facilitated the depolymerization of vimentin and promoted its accumulation in cytoplasm (Fig. S4A and B), it was considered that GP73 might play functional roles in vimentin polymerization.

Since it has been reported that polymerization of vimentin is regulated by the phosphorylation sites at S39, S56 and S83^20^, ^21^, phosphorylation status of vimentin was examined in the cell lines above. It demonstrated that GP73 facilitated dephosphorylation of vimentin at S56 and S83 to facilitate polymerization of vimentin-mediated intermediate filaments (Fig. 4B). Since S56 phosphorylation provides a PLK binding site to further phosphorylates vimentin at S83^21^, it is considered that GP73 regulates dephosphorylation of vimentin at S56 to promote vimentin polymerization.

As Serine/Threonine-protein phosphatase PP1-alpha (PP1A) was identified as an interaction partner of GP73 via LC-MS/MS and it was reported as a phosphatase facilitating dephosphorylation of vimentin at S56^22^, we considered that GP73 could be involved in the process of PP1A-mediated vimentin dephosphorylation at S56. Co-IP followed by immunoblotting manifested that PP1A could simultaneously interact with vimentin and GP73 (Fig. 4C). Notably, PP1A recruited more vimentin molecules in GP73 highly expressed cell lines, which suggested that the process of PP1A-mediated vimentin depolymerization was GP73-dependent. To elucidate the hypothesis, S56A and S56D mutants of vimentin were overexpressed in MHCC-97H cells and their interactions with GP73 were examined. It was revealed that GP73 could interact with vimentin dephosphorylated at S56 (Fig. 4D), indicating that GP73 might stabilize the vimentin molecules dephosphorylated by PP1A. Actually, the half-life of vimentin was prolonged in cells with highly expressed GP73 compared with those with low GP73 expression (Fig. 4E). Immunofluorescence staining further proved that GP73 could prolong vimentin-mediated intermediate filaments and GP73 was co-localized with S56A mutant of vimentin, which implied that GP73 might facilitate the polymerization of vimentin dephosphorylated at S56 (Fig. 4F). Native-PAGE and gel filtration assays further indicated that GP73 promoted polymerization of vimentin-mediated intermediate filaments (Fig. 4G and H). Besides, it was shown that knockdown of GP73 blocked polymerization in vimentin overexpressed HCC cells (Fig. 4I), which confirmed that GP73 was essential for vimentin polymerization.

Since GP73 interacted with vimentin dephosphorylated at S56 and facilitated vimentin polymerization, it was deemed that GP73 mediated polymerization of vimentin through vesicular trafficking. Normally, vesicular protein may share similar half-lives with its substrates^23^, Therefore, half-lives of GP73 and vimentin were determined by protein stability assay. The results demonstrated that there was no significant difference between the half-lives of GP73 and vimentin (Fig. S4C and D), reflecting that vimentin might act as a potential substrate of GP73-dependent vesicular trafficking. Besides, Brefeldin A (BFA) inhibited the formation of intracellular vesicles, resulting in vimentin depolymerized from intermediate filaments and facilitated its co-localization with GP73 in cytoplasm (Fig. S4E). Lastly, GP73-GFP and vimentin-OFP were co-expressed in 293T cells, followed with live-cell imaging using structural illumination microscopy. It was shown that intracellular vesicles containing GP73 crawled along intermediate filaments, indicating that GP73 directly participated in vimentin polymerization and intermediate filaments extension (Fig. 4J and Movie S1). In summary, GP73 promotes polymerization of vimentin to stabilize intermediate filaments in HCC cells.

### Clomipramine is identified as a specific inhibitor targeting vimentin polymerization

**Figure 5:**
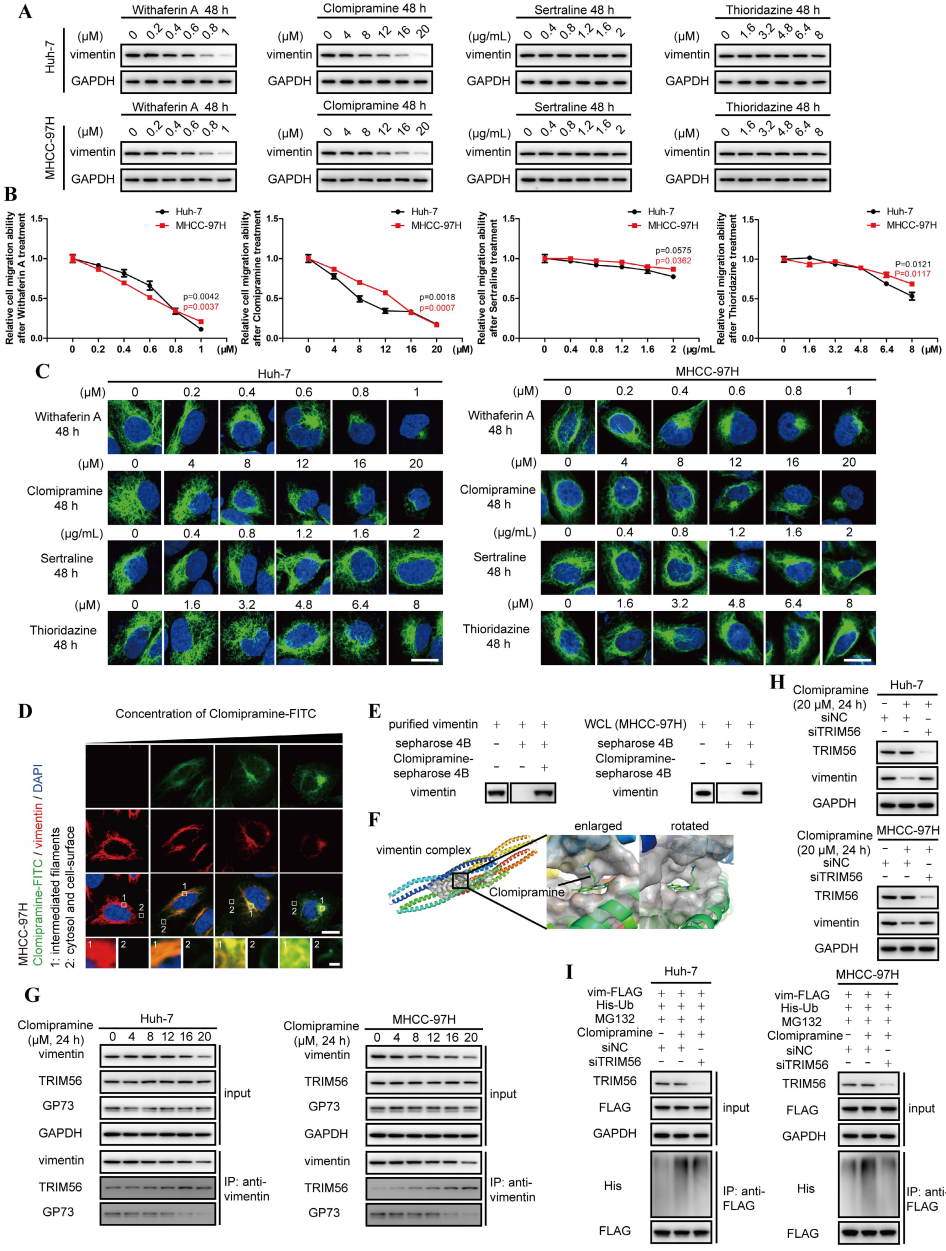
** Clomipramine is identified as a specific inhibitor targeting vimentin polymerization.** A. The level of vimentin was determined by immunoblotting analysis 48 h after Huh-7 and MHCC-97H cells were treated with Withaferin A, Clomipramine, Sertraline or Thioridazine as indicated. B. Relative cell migrative abilities of Huh-7 and MHCC-97H cells determined by Transwell migration assay 48 h after cells were planked onto Transwell chambers and treated with Withaferin A, Clomipramine, Sertraline or Thioridazine as indicated (n=3). C. Immunofluorescence staining of vimentin (green) 48 h after Huh-7 and MHCC-97H cells were treated with Withaferin A, Clomipramine, Sertraline or Thioridazine as indicated (scale bar: 5 μm). D. Immunofluorescence staining of Clomipramine-FITC (green) and vimentin (red) 48 h after MHCC-97 cells were treated with Clomipramine-FITC (0, 5, 10 and 20 μM, scale bar: 5 μm, upper; 500 nm, lower). E. Clomipramine-coupled Sepharose 4B beads were incubated with whole cells lysates of MHCC-97H or purified vimentin-FLAG fusion protein and Clomipramine binding proteins were eluted. Vimentin and vimentin-FLAG were examined using immunoblotting analysis. F. Putative binding site of Clomipramine and tetramers of vimentin coil1B domain. G. The interactions of vimentin/TRIM56 and vimentin/GP73 were determined using co-IP followed by immunoblotting 24 h after cells treated with Clomipramine as indicated concentrations. H. The level of vimentin was determined while cells transfected with siNC or siTRIM56 for 72 h. Cells were treated with Clomipramine (20 μM) 24 h before harvest. I. The ubiquitination level of vimentin-FLAG was determined while cells transfected with siNC or siTRIM56 for 72 h. Cells were treated with Clomipramine (20 μM) and MG132 (5 μM) 24 h before harvest. Data in B are the mean±s.e.m. and a two-tailed Student's *t*-test was used for statistical analysis.

Given the importance of vimentin polymerization to tumor metastasis, vimentin-specific inhibitors are potentially expected as valuable drugs in anti-cancer therapeutics. However, Withaferin A, the known vimentin-specific inhibitor has not been approved by Food and Drug Administration (FDA) for clinical applications^24^. It is worthwhile to screen potential inhibitors targeting vimentin from FDA-approval drugs. Some antidepressants including Clomipramine, Sertraline and Thioridazine, were predicted to interact with vimentin^25^-^28^. Meanwhile, only Clomipramine could effectively reduce the expression of vimentin, inhibit cell migration and promote vimentin depolymerization as Withaferin A (Fig. 5A-C, Fig. S5A and B). We therefore focused on Clomipramine in following studies.

Since 5HTR, the recognized target of Clomipramine, is little expressed in HCC cells, we tried to examine the co-localization of Clomipramine and vimentin (Fig. S5C and D), and it was discovered that FITC-tagged Clomipramine co-localized with vimentin in HCC cells (Fig. 5D). It was further proved that Clomipramine-conjugated Sepharose 4B beads could pull down vimentin from cell lysates (Fig. 5E and Fig. S5E). Furthermore, molecular docking predicted that Clomipramine interacted with coil1B domain of vimentin and formed steric hinderance to inhibit the formation of vimentin tetramers (Fig. 5F).

As a result, Clomipramine attenuated polymerization of vimentin tetramers and induced the accumulation of vimentin monomers, which shortened the half-lives of vimentin in HCC cells (Fig. S5F and G). Co-IP followed by immunoblotting indicated that Clomipramine facilitated the interaction of vimentin and TRIM56, but attenuated its interaction with GP73, which implied that Clomipramine might promote TRIM56-mediated vimentin degradation (Fig. 5G). Further study revealed that Clomipramine reduced the level of vimentin through facilitating its ubiquitin-dependent proteasome degradation, which was rescuable while TRIM56 knockdown (Fig. 5H and I).

Overall, Clomipramine is identified as a specific inhibitor targeting vimentin and facilitating its degradation.

### Clomipramine inhibits tumor metastasis and enhances the efficacy of Sorafenib *in vivo*

**Figure 6:**
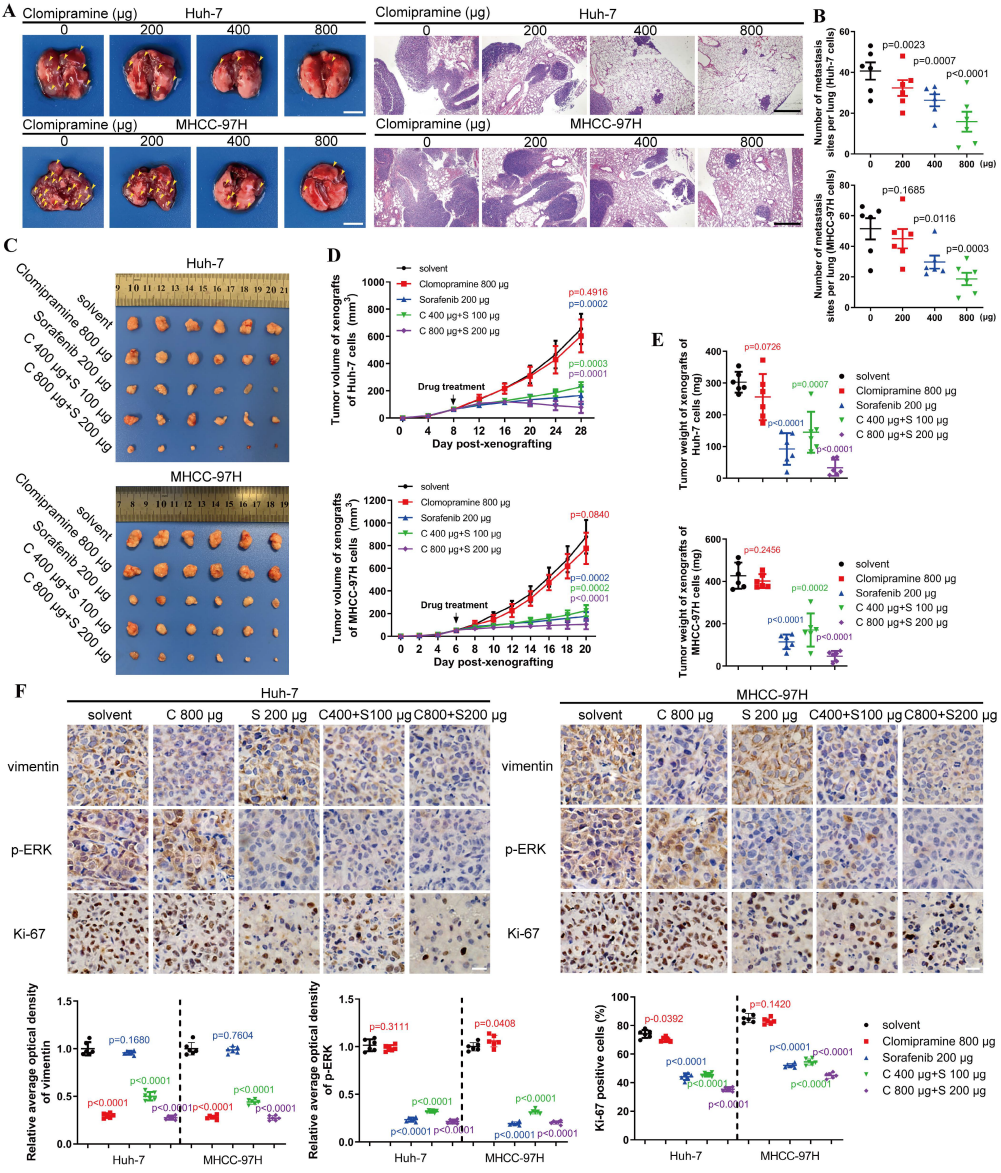
** Clomipramine inhibits tumor metastasis and enhances curative effect of Sorafenib *in vivo*.** A. Images of excised lung (scale bar: 5 mm) and H&E staining (scale bar: 1 mm) of lung metastases from nude mice (n=6 in each group). B. The lung metastasis sites from the lungs of nude mice were plotted (n=6). C. Images of tumors derived from nude mice bearing xenografts of Huh-7 and MHCC-97H cells (n=6 in each group). D. Tumor growth curves were plotted. Tumor sizes were measured every 4 d after mice were subcutaneously injected with HCC cells (n=6). E. The weights of tumors were measured immediately after they were excised and plotted (n=6). F. Immunohistochemical staining of vimentin, p-ERK and Ki67 in xenografts (scale bar: 10 μm). Data in B, D, E and F are the mean±s.e.m. and a two-tailed Student's *t*-test was used for statistical analysis.

An *in vivo* metastasis assay was performed to assess the anti-metastatic capacity and cytotoxicity of Clomipramine. As shown in Fig. 6A and B, lung metastasis of HCC cells was gradually inhibited as the dose of Clomipramine increased, and high dose of Clomipramine could significantly inhibit lung metastasis. Strikingly, Clomipramine impacted little to the weight of mice even in the high dose groups (Fig. S6A), proving a potential efficacy of Clomipramine in HCC metastasis with manageable toxicity in clinical practice.

As an effective and widely-used drug for HCC, Sorafenib is the first-line choice for the treatment of advanced HCC. However, the side-effects and drug resistance caused by EMT restricted the valid application of Sorafenib for HCC treatment^29^-^32^. More evidences have indicated that vimentin-mediated EMT directly induces sorafenib resistance^33^. Since Clomipramine is potentially expected as an anti-EMT drug targeting vimentin with low cytotoxicity, it is worth to explore the effect of Sorafenib combined with Clomipramine on HCC. We established xenograft mouse models using Huh-7 and MHCC-97H cells and treated with Sorafenib and Clomipramine. As expected, Sorafenib (200 μg/ml) was more effective against xenografts than Clomipramine (800 μg/ml) alone (Fig. 6C). However, tumor growth was dramatically inhibited once Sorafenib and Clomipramine were used in combination. Interestingly, combination of half dose of both Sorafenib and Clomipramine also revealed significant curative effect against tumor growth, similar with full dose Sorafenib group (Fig. 6D and E). In consistence with enhanced inhibition of tumor growth, Clomipramine promoted Sorafenib-induced inhibition of ERK, and cell proliferation was attenuated as indicated by Ki-67 staining (Fig. 6F). Notably, the side-effect as evidenced by weight loss was greatly relieved when Sorafenib was dosed in half, irrespective of the addition of Clomipramine (Fig. S6B). In conclusion, Clomipramine effectively reduced HCC metastasis and synergized with Sorafenib to inhibit HCC growth* in vivo*.

### Detection of serum GP73 serves as a potential approach for companion diagnosis of vimentin-high HCC

Since sGP73 has served as a biomarker for diagnosis of HCC and GP73 is positively associated with vimentin in HCC, it is worthwhile to further investigate whether sGP73 is a precise biomarker of companion diagnosis to select vimentin-high HCC for Clomipramine treatment. The specimens shown in Fig. 2B and the corresponding serum specimens were enrolled into cohort 1. Additionally, extra tissue and serum specimens (n=60) were collected and enrolled into cohort 2. Comparable clinical features and complete follow-up data were summarized in Table S1.

**Figure 7:**
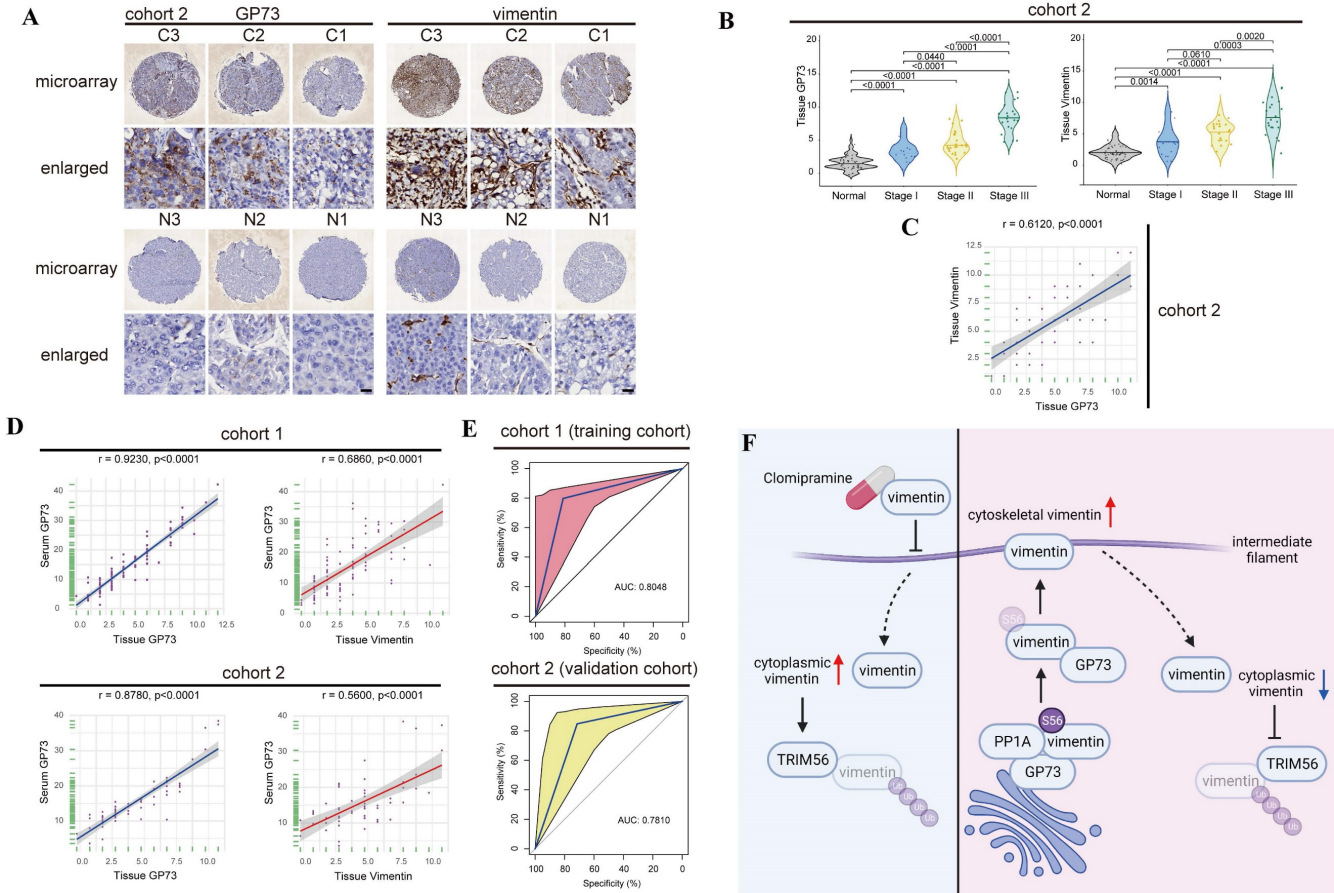
** Detection of serum GP73 serves as a potential approach for companion diagnosis of vimentin-high HCC.** A. Immunohistochemical analysis of GP73 and vimentin in primary tumor (n=60) and adjacent liver (n=60) tissues of HCC patients in Cohort 2. Images were labeled by staging of AJCC (1, 2 and 3) and tissue type (C=primary tumor, N=adjacent liver; scale bar: 10 μm). B. In cohort 2, the levels of GP73 and vimentin were shown as IHC scoring (n=60). C. In cohort 2, the correlation of GP73 and vimentin in primary tumor was represented (n=60). D. In cohort 1 and cohort 2, the correlations of serum GP73 / tissue GP73 and serum GP73 / tissue vimentin in serum and primary tumor tissues were represented (n=60). E. ROC analysis of serum GP73 in cohort 1 (training cohort) and cohort 2 (validation cohort) for the companion diagnosis of vimentin-high HCC (n=60). F. A brief illustration of the working model: GP73 facilitates EMT via stimulating polymerization of vimentin. Clomipramine has been identified as a novel inhibitor targeting vimentin polymerization and it enhances the effect of Sorafenib through inhibiting EMT. Serum GP73 serves as a potential biomarker for companion diagnosis of vimentin-high HCC. The image was created with BioRender.com. Data in B are the mean±s.e.m. and a two-tailed Student's *t*-test was used for statistical analysis, data in C and D are analyzed using spearman correlation analysis.

Similar with the result in cohort 1, GP73 and vimentin were highly expressed in primary tumor tissues in comparison with adjacent liver tissues in cohort 2 (Fig. 7A and B), and the level of GP73 protein was also significantly correlated with vimentin protein (r=0.6120, Fig. 7C). Furthermore, the levels of sGP73 in specimens from both cohorts were examined and they were highly correlated with the levels of GP73 in corresponding primary tumor tissues (cohort 1: r=0.9230; cohort 2: r=0.8780, Fig. 7D, Fig. S7A and B). Moreover, it was worth noting that sGP73 was significantly correlated with vimentin in primary tumor tissues (cohort 1: r=0.6860; cohort 2: r=0.5600, Fig. 7D), which implied that sGP73 might serve as a potential biomarker for companion diagnosis of vimentin-high HCC.

Thus, the receiver operating characteristic curve (ROC) analysis was performed to evaluate the performance of sGP73 in diagnosis of vimentin-high HCC. Cohort 1 were defined as the training cohort and cohort 2 was used as the validation cohort to validate the model established from the training cohort. The results revealed that the area under the curve (AUC) of sGP73 concentration to discriminate tissue vimentin IHC score was 0.8048 (95%CI: 0.6818-0.9280), with 81.3% of sensitivity and 80.0% of specificity in the training cohort (Fig. 7E). In the validation cohort, the AUC of sGP73 concentration to discriminate tissue vimentin IHC score was 0.7810 (95%CI: 0.6890-0.9331), with 71.4% of sensitivity and 84.8% of specificity (Fig. 7E). The best cut-off value of sGP73 concentration was 17.50 ng/mL, which could well discriminate vimentin expression in primary tumors of HCC patients.

All in all, data above have indicated that sGP73 potentially serves as a serum biomarker for companion diagnosis of vimentin-high metastatic HCC, which might be an indication for Clomipramine application.

## Discussion

As a cytoskeletal protein in the family of type Ⅲ intermediate filaments, vimentin is originally expressed in mesenchymal cells but upregulated during cancer metastasis^34^, ^35^. The vimentin network is involved in organelle positioning, cell migration, signal transduction and, particularly, it maintains mechanical resistance of cancer cells^36^. The expression of vimentin was usually increased during tumorigenesis featured by EMT. Tetramerized vimentin molecules associate with keratins and desmosomes at the karyotheca and cell periphery, and extension of vimentin-mediated intermediate filaments maintains the shape of mesenchymal cells to facilitate HCC metastasis^37^. In this study, we found that GP73 interacted with vimentin to prevent it from TRIM56-medicated polyubiquitination and subsequent proteasomal degradation, thus facilitating tumor metastasis. It has been discovered that GP73 facilitates proliferation and metastasis of tumor cells through regulating the expressions and functions of multiple cell growth and metastasis-associated oncoproteins. As a Golgi-resident protein, GP73 was believed to facilitate the trafficking of membrane proteins such as EGFR or secretory proteins including MMP-2 and MMP-7. Herein, we found that GP73 promotes the assembly of vimentin intermediate filament network by promoting the translocation of vimentin from the cytoplasm to cytoskeleton and it prevents TRIM56-mediated polyubiquitination and proteasomal degradation of cytoplasmic vimentin (Fig. 7F). During the assembly of vimentin intermediate filament, vimentin monomers initially form parallel dimers that in turn assemble into tetramers in a staggered manner^34^, ^38^. Eight tetramers further polymerize into 'unit-length filaments' that connect head to tail and compact to give the 10 nm-wide mature filaments^39^, ^40^. We found that TRIM56 interacts with vimentin monomers in cytoplasm but not tetramers on cytoskeleton since the lysine ubiquitinated by TRIM56 buried inside once polymerized. GP73 decreases the level of vimentin monomers in cytoplasm so as to reduce the interaction of vimentin with TRIM56, thus extending the half-life of vimentin and increasing its expression. The dynamic vimentin polymerization and intermediate filament assembly could be regulated by various post-translational modifications such as nitrosylation and interactions with other cytoskeletal or scaffold proteins^34^, ^41^. However, it remains unknown whether post-translational modifications of vimentin affect its interaction with GP73.

As a functional consequence of vimentin polymerization triggered by GP73, the motility of HCC cells is promoted once the expression of GP73 was upregulated. Actually, it has been reported that GP73 facilitates HCC cell migration *in vitro* and lung metastasis *in vivo*, which is rescuable while GP73 was silenced^7^, ^8^. The studies above further evidence that cell motility induced by GP73 might be vimentin polymerization dependent. Besides, high expression of either GP73 or vimentin was associated with poor prognosis of HCC patients, therefore, targeting GP73-mediated vimentin polymerization could be provided as a novel strategy for the treatment of metastatic HCC. Unfortunately, no FDA-approved drugs targeting vimentin were clinically available. After screening drugs in FDA-approved drugs database, Clomipramine, the anti-depressant was identified as a potential vimentin inhibitor. Our following studies indicated that Clomipramine effectively blocked vimentin polymerization, leading cell migration and tumor metastasis inhibited. Besides, it was discovered that Clomipramine enhanced the anti-cancer effect of Sorafenib *in vivo* through inhibiting sorafenib-induced EMT. While Sorafenib has established its fundamental role in the targeted therapy of HCC, EMT contributed to the primary or acquired resistance of HCC to Sorafenib. By inhibiting vimentin polymerization, Clomipramine could be of potential value to overcome or prevent Sorafenib resistance with tolerable side effects in the clinical management of HCC.

For precision treatment nowadays, any effective treatments should be applied under the guidance of companion diagnoses. As advanced HCC patients are not suitable for surgery even frequent biopsies, liquid biopsy represents one ideal approach of companion diagnosis to direct target therapy of interest. It has indicated that the level of intracellular vimentin positively correlates with that of intracellular GP73, which is also significantly correlated with sGP73 since the secretion of GP73 is constitutive. As a result, sGP73 has succeeded to reflect the expression of vimentin in HCC tissues. Clinical trials to evaluate the value of sGP73 to predict Clomipramine response of certain HCC patients are warranted in the future.

In summary, GP73 interacts with vimentin and promotes its polymerization to intermediate filaments, which inhibits TRIM56-mediated degradation of vimentin monomers. Clomipramine, as an anti-depressant, impairs vimentin polymerization to effectively inhibit metastasis of HCC with highly expressed GP73 and enhances the effect of Sorafenib for HCC treatment *in vivo*. Furthermore, detection of sGP73 could serve as a biomarker for companion diagnosis to select GP73-high HCC for Clomipramine application.

## Supplementary Material

Supplementary methods, figures and tables, movie legend.

Supplementary movie.

## Abbreviations

GP73: Golgi-protein 73
HCC: hepatocellular carcinoma
PP1A: Serine/Threonine-protein phosphatase PP1-alpha
5-HTR: 5-hydroxytryptamine receptor
sGP73: serum GP73
HBV: hepatitis B virus
HCV: hepatitis C virus
EMT: epithelial-mesenchymal transition
EGFR: epidermal growth factor receptor
PD-L1: programmed cell death-ligand1
MMP: matrix metalloproteinase
co-IP: co-immunoprecipitation
OS: overall survival
SIM: structural illumination microscopy
BFA: Brefeldin A
FDA: Food and Drug Administration
ROC: receiver operating characteristic curve
AUC: area under the curve
