# Supplementary material for: Golgi-protein 73 facilitates vimentin polymerization in hepatocellular carcinoma
Source: Int J Biol Sci. 2023 Jul 16;19(12):3694–708. doi: 10.7150/ijbs.85431 (PMC10411459; doi:10.7150/ijbs.85431)
Supplement: Supplementary file 1 — Supplementary methods, figures and tables, movie legend. [file ijbsv19p3694s1.pdf]

Supplementary materials

Supplementary materials and methods

Plasmid and siRNA transfection

The plasmids pCMV3-GP73-C-FLAG, pCMV3-GP73, pCMV3-GP73-GFP, pCMV3-vimentin-OFP, pCMV3-vimentin, pCMV3-vimentin-C-FLAG and pCMV3-TRIM56 were purchased from Sinobiological (Beijing, China). Truncated mutants of GP73 and vimentin were constructed based on pCMV3-GP73-FLAG and pCMV3-vimentin-FLAG. The siRNAs corresponding to human GP73 (*GOLM1*), TRIM16 (*TRIM16*), gigaxonin (*GAN*), TRIM56 (*RNF109*) and vimentin (*VIM*) were purchased from GenePharma (Shanghai, China). Details on the siRNAs are shown in Table S2. Transfection of plasmids and siRNAs were performed using Lipofectamine 3000 (plasmids) and Lipofectamine RNAiMax (siRNAs) reagents (Thermo Fisher, Carlsbad, CA, USA) following the manufacturer's instructions.

RNA isolation and Quantitative real-time PCR

Total RNA was isolated using a TaKaRa MiniBEST Universal RNA Extraction Kit (TaKaRa, Dalian, China). Genomic DNA was eliminated and cDNA first strands were synthesized using a PrimeScript™ RT reagent Kit with gDNA Eraser (TaKaRa) following the manufacturer's instructions. The levels of target mRNAs were quantified via qRT-PCR using a CFX-96 Real-Time PCR system (Bio-Rad, Hercules, CA, USA). qRT-PCR was conducted using SYBR Premix Ex Taq® reagent (TaKaRa) with the primers listed in Table S3. The mRNA level of β-actin was used as a reference.

Co-immunoprecipitation analysis

Cells were lysed using 1×IP lysis buffer (Thermo Fisher) with a protease and phosphatase inhibitor cocktail for 30 m, and centrifuged at 15,000×*g* for 15 m. The supernatants were collected and incubated with the mixture of antibodies and Protein A/G magnetic beads (Thermo Fisher) for 6 h at 4℃ with gently shaking. Proteins were eluted by IP elution buffer (0·1M glycine, pH=2·0). For purification of exogenous proteins with FLAG-tag or HA-tag, proteins were co-immunoprecipitated using an Anti-DYKDDDDK Magnetic Agarose Kit (Thermo Fisher) or Anti-HA Magnetic Beads (Thermo Fisher) and eluted using 3×DYKDDDDK peptides (Thermo Fisher) or HA synthetic peptide (Thermo Fisher) followed with manufacturer's instructions. Target proteins and their interacted proteins were examined using immunoblotting analysis or mass spectrum.

Immunoblotting analysis

Cells were lysed using RIPA lysis buffer (Merck-Millipore, Darmstadt, Germany) with a protease and phosphatase inhibitor cocktail (Thermo Fisher). Concentrations of lysates were determined using a BCA protein quantification kit (Beyotime, Nanjing, China) and samples were subjected to immunoblotting as previously described[1]. Details on the antibodies are shown in Table S4.

H&E and immunohistochemical staining

Paraffin embedded sections of xenografts were manufactured in our previous study[1]. H&E and immunohistochemical staining were performed as previously reported[1]. Details on the antibodies are shown in Table S4.

Transwell migration assay

For determination of migrative abilities of Hep3B and MHCC-97H cells after GP73 mediation, cells were harvested 48 h after transfection and chamber was planked with 30,000 cells diluted in 100 μL serum-free DMEM. The bottom of the well was filled with 800 μL DMEM with 10% FBS. Cells were incubated in 5% CO_2 at 37°C for extra 24 h and fixed with methanol (>99·.5%) for 30 m. Cells were stained with 0·3% crystal violet solution for 2 h and cells in the upper chamber were removed. Images were captured using an Olympus DP70 microscope in the bright field (Olympus Corporation, Tokyo, Japan). For determination of migrative abilities of Huh-7 and MHCC-97H cells, chamber was planked with 20,000 cells as described above. Culture media in both chamber and plate was mixed with drugs as indicated. Cells were cultured for extra 48 h instead of 24 h.

Mapping of the binding sites of GP73/vimentin and vimentin/TRIM56 *in vitro*

Truncated mutants of GP73 and vimentin were designed, as shown in Fig. 1d and 2f. Mutants were constructed based on the pCMV3-GP73-FLAG and pCMV3-vimentin-FLAG plasmids. Protein extraction, co-IP and immunoblotting were performed as described above.

Isolation of cytoskeletal and cytoplasmic components

Cytoskeletal and cytoplasmic (without nuclear and cell-surface components) components were isolated using a Subcellular Protein Fractionation Kit for Cultured Cells (Thermo Fisher) according to the manufacturer's instructions. Protein concentrations of cytoskeletal and cytoplasmic extracts were determined using a BCA protein quantification kit (Beyotime, Nanjing, China). α-actinin and GAPDH were used as internal references of cytoskeletal and cytoplasmic proteins.

Immunofluorescence staining and confocal microscopy

Immunofluorescence staining was performed as previously reported[1]. Details on the antibodies are shown in Table S4. Images were captured using an FV3000 confocal microscope (Olympus Corporation, Tokyo, Japan). For identification of fine structures of vimentin-mediated intermediated filaments, images were captured using a structural illumination microscope (SIM, Nikon Corporation, Japan).

Prediction of structure of TRIM56-vimentin complex

The predicted structures of TRIM56 (Q9BRZ2) and vimentin (P08670) were predicted using AlphaFold 2·0 software and obtained from AlphaFold Protein Structure Database (https://alphafold.ebi.ac.uk). The RING domain of TRIM56 was validated using crystal structure of SopA-TRIM56 complex (PDB accession number: 5JW7) and the coil domains of vimentin were validated using crystal structures of vimentin coil1A, coil1B and coil2 (PDB accession number: 1GK7, 3SWK and 3TRT). Putative structures of TRIM56-vimentin complex were calculated using GRAMM-X Protein-Protein Docking Web Server v.1·2·0. Predicted candidate complex molecules were selected according to the results of mapping of vimentin and TRIM56. Notably, the lysine residue should be dropped in RING domain of TRIM56. The selected structure was validated via mutation of predicted ubiquitination site of vimentin and determination of ubiquitination of wild-type vimentin and vimentin mutant.

Native-PAGE analysis of vimentin

Cells were collected and resuspended in cold PBS (pH=8.0) and DSS (disuccinimidyl substrate) solution (Thermo Fisher) was added to the final concentration of 1 mM. The reaction mixture was incubated for 30 m at room temperature and the reaction was stopped by adding quench solution (1 M TRIS, pH=7.5) to the final concentration of 10 mM TRIS. Cells were lysed using IP lysis buffer (Thermo Fisher) followed with the manufacturer's instructions and mixed with Native Gel Sample Loading Buffer (Beyotime). Samples were separated using a BeyoGel Blue Native PAGE precast gel (Beyotime) and detected using immunoblotting as described above.

Preparation of FITC and Sepharose 4B modified Clomipramine

Fluorescein 5-isothiocyanate (FITC) and Sepharose 4B were linked to Clomipramine as shown in Fig. S4d and e.

Supplementary figures

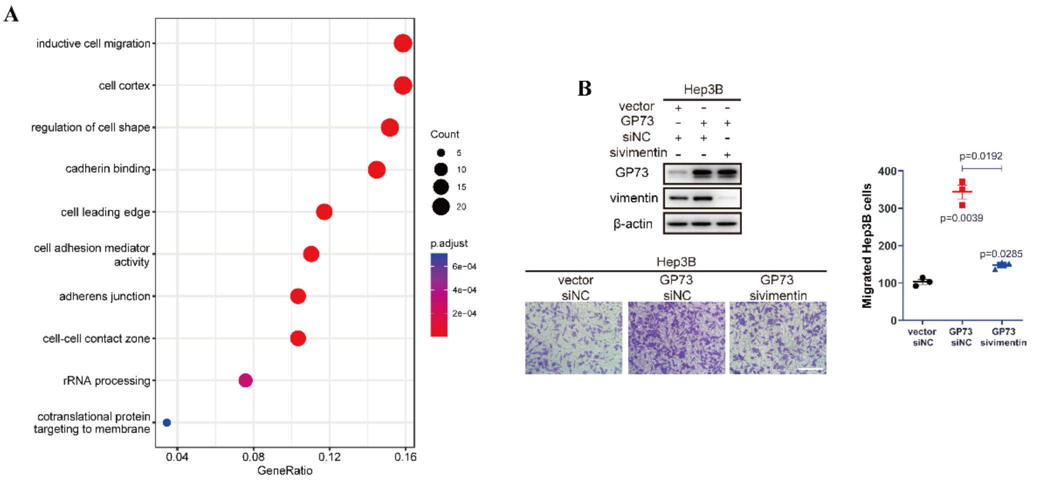

Figure S1. Vimentin is identified as a new GP73-interacting protein facilitating HCC migration.

A. Gene Ontology (GO) analysis indicated that GP73-interacting proteins were closely associated with cell migration.

B. The levels of GP73 and vimentin were determined in HepG2 cells transfected with siRNAs and vectors as described for 72 h. Transwell migration assay was conducted as above (n=3, scale bar: 100 µm).

Data in B are the mean±s.e.m. and a two-tailed Student's *t*-test was used for statistical analysis.

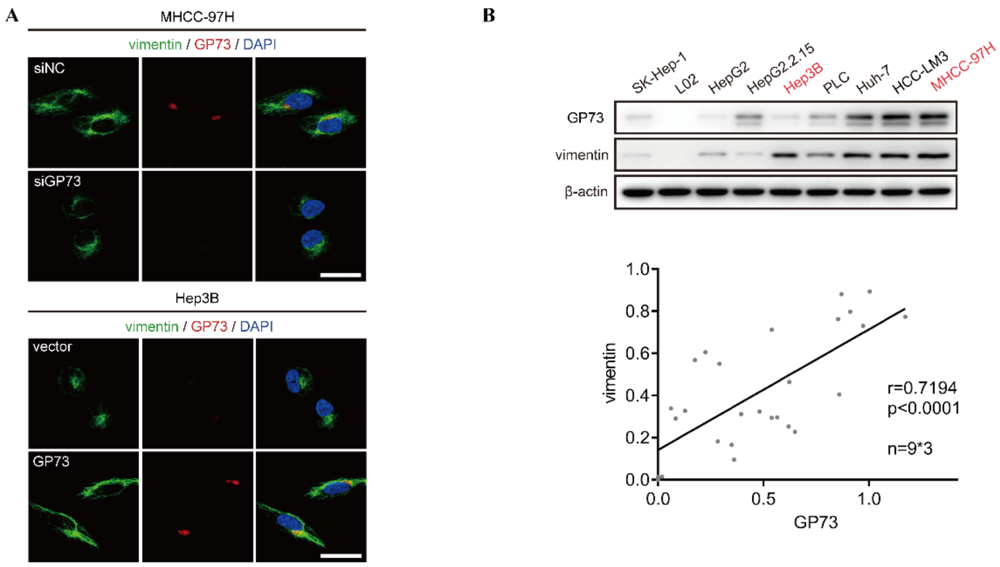

Figure S2. GP73 positively regulates the expression of vimentin.

A. Immunofluorescence staining and confocal microscopy of vimentin (green) and GP73 (red) in MHCC-97H and Hep3B cells 72 h after the level of GP73 was modulated as indicated (scale bar: 10 µm).

B. The levels of GP73 and vimentin in normal liver and HCC cell lines were determined by immunoblotting analysis. The correlation of GP73 and vimentin in indicated cell lines was represented using linear correlation.

Data in B are analyzed using spearman correlation analysis.

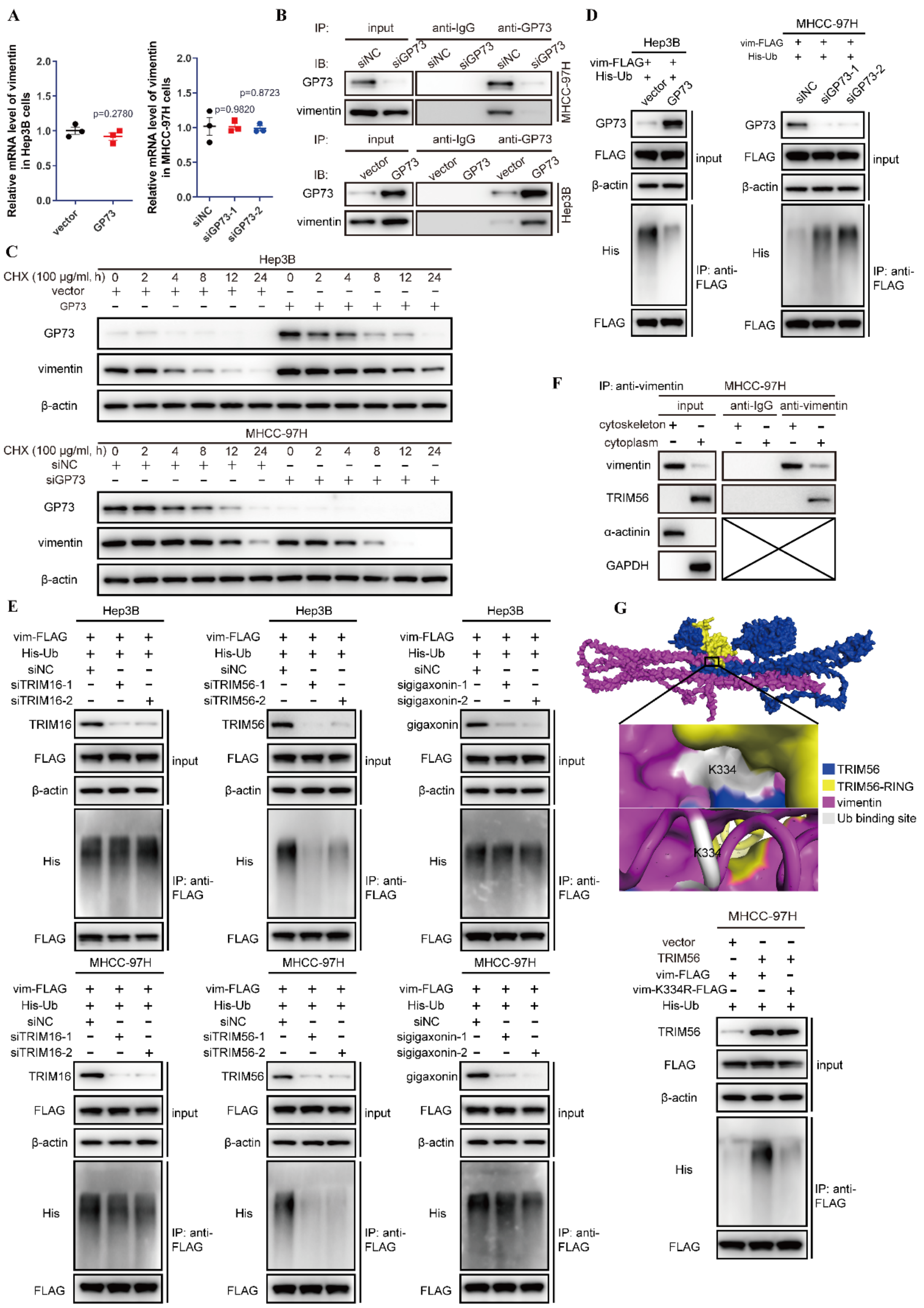

Figure S3. GP73 stabilizes vimentin by preventing its polyubiquitination mediated by TRIM56.

A. The mRNA level of vimentin was determined by qRT-PCR 72 h after the level of GP73 in MHCC-97H and Hep3B cells were modulated as indicated.

B. The interaction of GP73 and vimentin was determined by immunoblotting analysis followed with co-IP 72 h after the level of GP73 mediated in MHCC-97H and Hep3B cells.

C. The levels of GP73 and vimentin were determined by immunoblotting analysis after GP73 was

mediated in Hep3B and MHCC-97H cells for 72 h. Cells were treated with CHX (100 μg/mL) for 0, 2, 4, 8, 12 and 24 h before cells were harvested.
D. The ubiquitination level of vimentin-FLAG was determined by immunoblotting analysis 72 h after the level of GP73 mediated in MHCC-97H and Hep3B cells.
E. The ubiquitination level of vimentin-FLAG was determined by immunoblotting analysis 72 h after the levels of indicated E3 ubiquitin-protein ligases were mediated in MHCC-97H and Hep3B cells.
F. Cytoskeletal and cytoplasmic components of MHCC-97H cells were isolated and the interaction of vimentin and TRIM56 was examined by immunoblotting analysis followed with co-IP.
G. Predicted structure of vimentin-TRIM56 complex and ubiquitination site of vimentin (K334) ubiquitinated by TRIM56 was shown. The model was verified using immunoblotting analysis.
Data in A are the mean±s.e.m. and a two-tailed Student's *t*-test was used for statistical analysis.

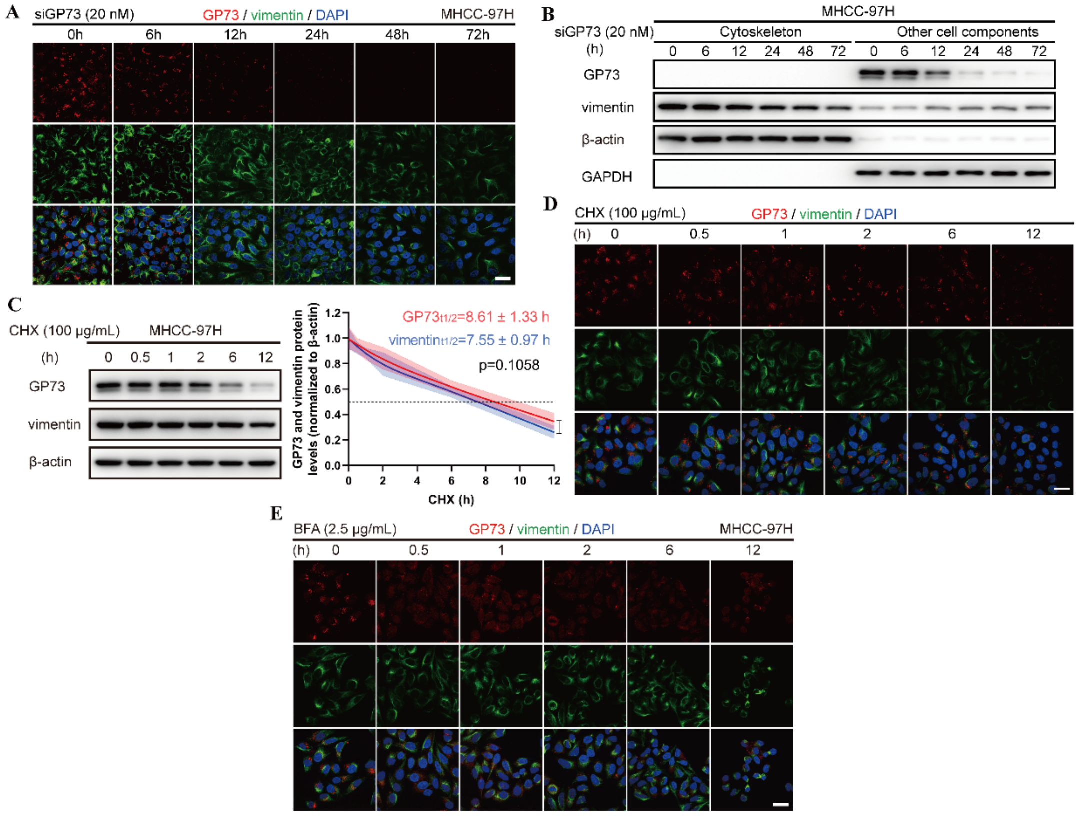

Figure S4. GP73 promotes polymerization of vimentin to stabilize intermediate filament network.

A. Immunofluorescence staining of GP73 (red) and vimentin (green) after MHCC-97H cells were transfected with siGP73 (20 nM) for 0, 6, 12, 24, 48 and 72 h (scale bar: 10 μm).

B. The levels of GP73 and vimentin in cytoskeleton and other cell components were determined by immunoblotting analysis after MHCC-97H cells were transfected with GP73-specific siRNA (20 nM) for 0, 6, 12, 24, 48 and 72 h.

C. The levels of GP73 and vimentin were determined by immunoblotting analysis after MHCC-97H cells were treated with CHX (100 μg/mL) for 0, 0·5, 1, 2, 6 and 12 h.

D. Immunofluorescence staining of GP73 (red) and vimentin (green) after MHCC-97H cells were treated with CHX (100 μg/mL) for 0, 0·5, 1, 2, 6 and 12 h (scale bar: 10 μm).

E. Immunofluorescence staining of GP73 (red) and vimentin (green) after MHCC-97H cells were treated with BFA (2·5 μg/mL) for 0, 0·5, 1, 2, 6 and 12 h (scale bar: 10 μm).

Data in C are the mean±s.e.m. and a two-tailed Student's *t*-test was used for statistical analysis.

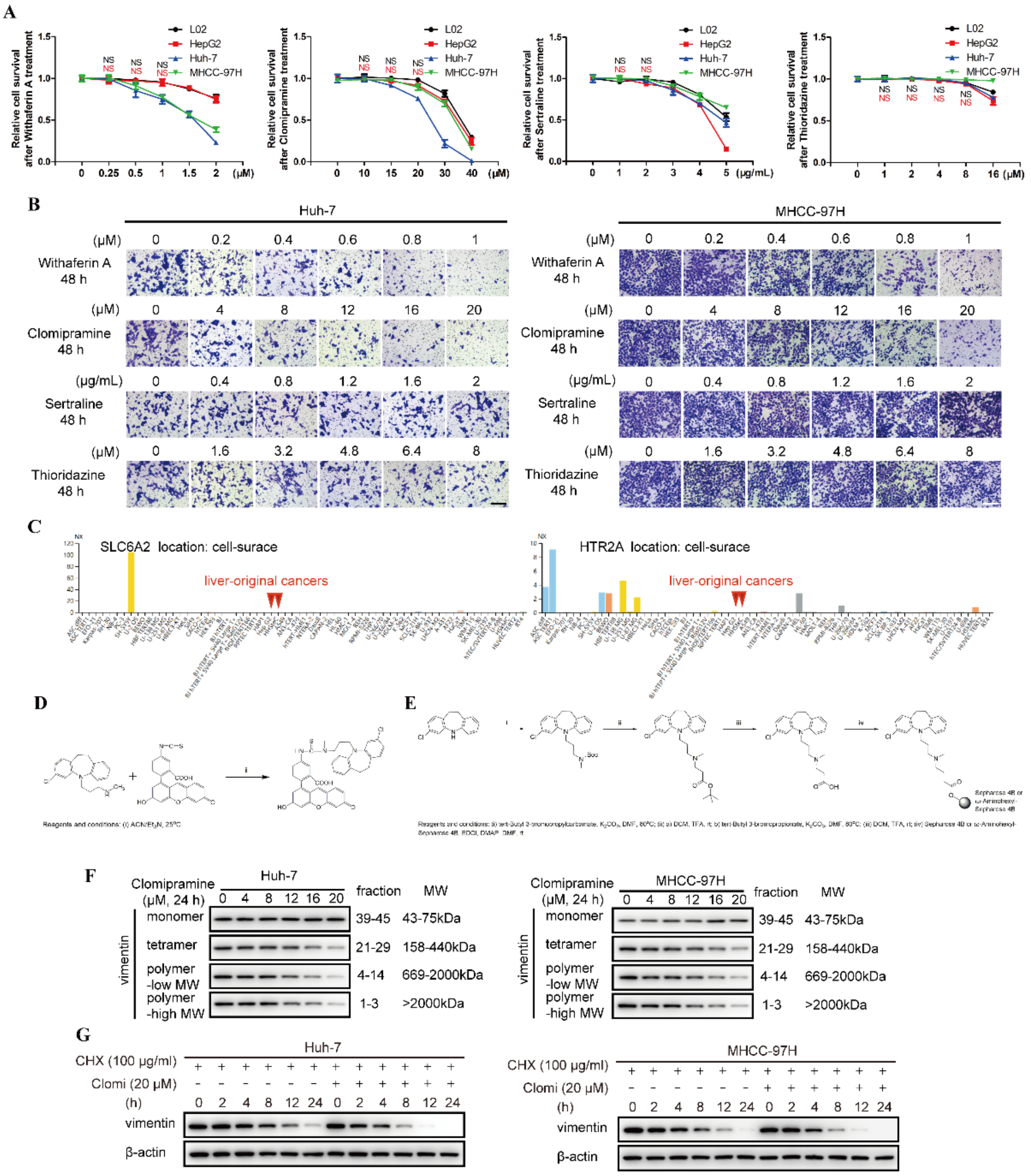

Figure S5. Clomipramine is identified as a specific inhibitor targeting vimentin polymerization.

A. Survival curves of L02, HepG2, Huh-7 and MHCC-97H cells after cells were treated with Withaferin, Clomipramine, Sertraline or Thioridazine as indicated concentrations for 48 h.

B. Huh-7 and MHCC-97H cells were planked into Transwell chambers and treated with Withaferin, Clomipramine, Sertraline or Thioridazine as indicated concentrations for 48 h. Then, migrated cells were stained using crystal violet and counted (scale bar: 100 µm).

C. The levels of SLC6A2 and HTR2A, the known targets of Clomipramine, in liver-origin cancers.

D. The synthetic procedures of Clomipramine-FITC.

E. The synthetic procedures of Sepharose 4B-coupled Clomipramine.

F. Components in whole cell lysates of Huh-7 and MHCC-97H cells were separated by gel filtration as molecular weights after cells were treated with Clomipramine (0, 4, 8, 12, 16 and 20 µM) for 24 h and the level of vimentin in each component were determined by immunoblotting analysis.

G. The level of vimentin was determined by immunoblotting analysis 0, 2, 4, 8, 12 and 24 h after Huh-7 and MHCC-97H cells were treated with CHX (100 µg/mL) and Clomipramine (20 µM) as indicated.

Data in A are the mean±s.e.m. and a two-tailed Student's *t*-test was used for statistical analysis.

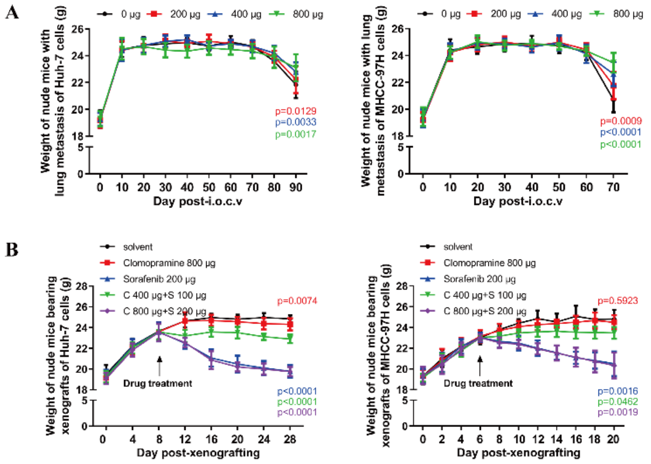

Figure S6. Clomipramine inhibits tumor metastasis and enhances curative effect of Sorafenib *in vivo*.

A. Body weight curves of nude mice with lung metastasis of HCC cells were plotted. Body weights were measured every 10 d after mice were injected with HCC cells via tail veins.

B. Body weight curves of nude mice bearing xenografts of HCC cells were plotted. Body weights were measured every 2 d after mice were subcutaneously injected with HCC cells.

Data in A and B are the mean±s.e.m. and a two-tailed Student's *t*-test was used for statistical analysis.

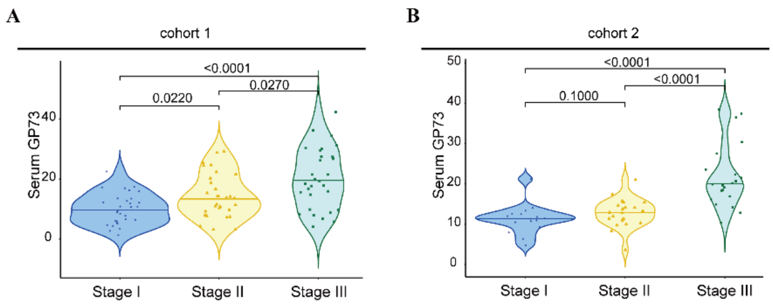

Figure S7. The level of serum GP73 in serum derived from HCC patients.

A. The comparison of serum GP73 concentration in different clinical stages of HCC patients in cohort 1.

B. The comparison of serum GP73 concentration in different clinical stages of HCC patients in cohort 2.

Data in A and B are the mean±s.e.m. and a two-tailed Student's *t*-test was used for statistical analysis.

Movie S1. The dynamics of internalized GP73-GFP, in green, and vimentin-OFP, in orange-red, structures in live cells.

Supplementary tables

Table S1. Demographic and clinical data of study population.

	Cohort 1 (n=90)	Cohort 2 (n=60)	Combined Cohort (n=150)
**Gender, n (%)**			
**Male**	72 (80.0)	53 (88.3)	125 (83.3)
**Female**	18 (20.0)	7 (11.7)	25 (16.7)
**HBV infection, n (%)**			
**Negative**	15 (16.7)	11 (18.3)	26 (17.3)
**Positive**	75 (83.3)	49 (81.7)	124 (82.7)
**Clinical stage, n (%)**			
**I**	30 (33.3)	16 (26.7)	46 (30.7)
**II**	30 (33.3)	23 (38.3)	53 (35.3)
**III**	30 (33.4)	21 (35.0)	51 (34.0)
**GP73 IHC score, median (IQR)**			
**Tumor tissue**	4.0 (2.0, 6.0)	5.0 (3.3, 8.0)	4.0 (3.0, 7.0)
**Normal tissue**	1.0 (1.0, 2.0)	1.0 (1.0, 2.0)	1.0 (1.0, 2.0)
**Vimentin IHC score, median (IQR)**			
**Tumor tissue**	4.0 (3.0, 6.0)	6.0 (4.0, 7.0)	4.5 (3.0, 6.0)
**Normal tissue**	1.5 (1.0, 2.0)	2.0 (1.0, 3.0)	2.0 (1.0, 2.0)
**Serum GP73 Concentration, ng/mL, median (IQR)**	13.54 (7.99,20.04)	13.90 (11.29, 18.97)	13.61 (9.76, 19.45)

Table S2. Sequences of siRNAs.

Targets	Sense and antisense chains (5’-3’)
**siGP73-1**	S: GAACAGUGUGAGGAGCGAATT
	A: UUCGCUCCUCACACUGUUCTT
**siGP73-2**	S: GUUGAGAAAGAGGAAACCATT
	A: UGGUUUCCUCUUUCUCAACTT
**siTRIM16-1**	S: GGUGAACAUCAAACUGCAATT
	A: UUGCAGUUUGAUGUUCACCTT
**siTRIM16-2**	S: GCAAAUAUUGGACUUCCAATT
	A: UUGGAAGUCCAAUAUUUGCTT
**sigigaxonin-1**	S: GAGUGAGUUCCUCCAGUUATT
	A: UAACUGGAGGAACUCACUCTT
**sigigaxonin-2**	S: GAGAGAGAUCCUGGAUUACTT
	A: GUAAUCCAGGAUCUCUCUCTT
**siTRIM56-1**	S: GCAGCAGAAUAGUGUGGUAAUTT
	A: AUUACCACACUAUUCUGCUGCTT
**siTRIM56-2**	S: UGUGGAUAAGAAGGGCUACAUTT
	A: AUGUAGCCCUUCUUAUCCACATT
**sivimentin**	S: GCAGGAUGAGAUUCAGAAUAUTT
	A: AUAUUCUGAAUCUCAUCCUGCTT
**siNC**	S: GCGACGAUCUGCCUAAGAUTT
	A: AUCUUAGGCAGAUCGUCGCTT

Table S3. Sequences of primers used in RT-qPCR.

Gene	Primer sequences (5’-3’)
GOLM1	F: CAGCGCTGATTTTGAGATGAC
	R: ATGATCCGTGTCTGGAGGTC
VIM	F: GGACCAGCTAACCAACGACA
	R: AAGGTCAAGACGTGCCAGAG
ACTB	F: TTCCAGCCTTCCTTCCTGGG
	R: TTGCGCTCAGGAGGAGCAAT

Table S4. Antibodies used for Western blotting, immunofluorescence and immunohistochemical analyses.

Antibody	Company	Cat. NO.	Dilution
GOLPH2 (GP73)	Thermo Fisher	PA5-30622	1:3000 WB
Vimentin	Cell Signaling Technology	5741S	1:3000 WB
α-actinin (D6F6)	Cell Signaling Technology	6487S	1:2000 WB
GAPDH (D16H11)	Cell Signaling Technology	5174S	1:3000 WB
TRIM16	Abcam	ab72129	1:2000 WB
TRIM56	Abcam	ab154862	1:2000 WB
GAN (gigaxonin)	Proteintech	14305-1-AP	1:5000 WB
6*His-Tag	Proteintech	66005-1-Ig	1:1000 WB
DYKDDDDK Tag	Cell Signaling Technology	14793S	1:3000 WB
HA-Tag	Cell Signaling Technology	3724S	1:2000 WB
β-actin	EarthOX	E021020-01	1:10000 WB
Vimentin (S39)	Cell Signaling Technology	13614S	1:2000 WB
Vimentin (S56)	Cell Signaling Technology	3877S	1:2000 WB
Vimentin (S83)	Cell Signaling Technology	3878S	1:2000 WB
PP1A	Cell Signaling Technology	2582S	1:4000 WB
GOLM1 (GP73)	Abnova	H00051280-M04	1:200 IP
Vimentin	Abcam	ab137321	1:100 IP
GOLM1 (GP73)	Abnova	H00051280-B01P	1:400 IF
Vimentin	Cell Signaling Technology	5741S	1:200 IF
GOLPH2 (GP73)	Thermo Fisher	PA5-30622	1:1000 IHC
Vimentin	Cell Signaling Technology	5741S	1:200 IHC
p-ERK (T202/Y204)	Cell Signaling Technology	4370S	1:500 IHC
Ki-67 (D2H10)	Cell Signaling Technology	9027S	1:800 IHC

References

1. Liu Y, Zhou S, Shi J, *et al*. c-Myc transactivates GP73 and promotes metastasis of hepatocellular carcinoma cells through GP73-mediated MMP-7 trafficking in a mildly hypoxic microenvironment. Oncogenesis 2019;8:58.
